# Supplementary material for: Benchmarking resting state fMRI connectivity pipelines for classification: robust accuracy despite processing variability in cross-site eye state prediction
Source: Brain Inform. 2026 May 5;13(1):18. doi: 10.1186/s40708-026-00305-1 (PMC13197499; doi:10.1186/s40708-026-00305-1)
Supplement: Supplementary file 1 — Supplementary Material 1 [file 40708_2026_305_MOESM1_ESM.pdf]

Supplementary materials present extended results including full-sized tables and figures for all datasets analyzed across sites, denoising strategies, atlases, and connectivity metrics.

QC-FC Results

Top-20 results according to QC-FC score are shown in Table S1 for EO and Table S2 for EC (ranking was based on lowest absolute correlation with motion).

Table S1. Top-20 pipelines for EO according to QC-FC score

Site	Atlas	Strategy	GSR	FC	QC_FC
ihb	Schaefer200	a/tCompCor(50%)+24P	noGSR	glasso	0.0784
ihb	AAL	a/tCompCor(50%)+24P	noGSR	glasso	0.0786
ihb	Brainnetome	a/tCompCor(50%)+24P	noGSR	glasso	0.0793
ihb	Schaefer200	aCompCor(50%)+12P	noGSR	glasso	0.0795
ihb	Schaefer200	aCompCor(50%)+24P	noGSR	glasso	0.0795
ihb	Brainnetome	aCompCor(50%)+12P	noGSR	glasso	0.0798
ihb	Schaefer200	aCompCor+12P	noGSR	glasso	0.0803
ihb	Schaefer200	aCompCor+24P	noGSR	glasso	0.0804
ihb	Schaefer200	AROMA Non-Aggressive	noGSR	glasso	0.0804
ihb	Brainnetome	aCompCor+12P	noGSR	glasso	0.0806
ihb	Brainnetome	aCompCor(50%)+24P	noGSR	glasso	0.0807
ihb	Brainnetome	24P	noGSR	glasso	0.0807
ihb	Schaefer200	24P	noGSR	glasso	0.081
ihb	Brainnetome	AROMA Non-Aggressive	noGSR	glasso	0.0812
ihb	Brainnetome	aCompCor+24P	noGSR	glasso	0.0814
ihb	Brainnetome	aCompCor(50%)+12P	GSR	glasso	0.0816
ihb	AAL	aCompCor(50%)+24P	noGSR	glasso	0.0819
ihb	Schaefer200	aCompCor(50%)+12P	GSR	glasso	0.082
ihb	Schaefer200	AROMA Non-Aggressive	GSR	glasso	0.0821
ihb	Brainnetome	24P	GSR	glasso	0.0822

Table S2. Top-20 pipelines for EC according to QC-FC score

Site	Atlas	Strategy	GSR	FC	QC_FC
ihb	AAL	a/tCompCor(50%)+24P	noGSR	glasso	0.0817
ihb	Brainnetome	aCompCor(50%)+24P	noGSR	glasso	0.0825
ihb	Brainnetome	a/tCompCor(50%)+24P	noGSR	glasso	0.0826
ihb	Brainnetome	aCompCor(50%)+12P	noGSR	glasso	0.0827
ihb	AAL	a/tCompCor(50%)+24P	GSR	glasso	0.0827
ihb	Brainnetome	AROMA Non-Aggressive	noGSR	glasso	0.083
ihb	Schaefer200	a/tCompCor(50%)+24P	noGSR	glasso	0.0831
ihb	Schaefer200	AROMA Non-Aggressive	noGSR	glasso	0.0832
ihb	AAL	a/tCompCor(50%)+24P	noGSR	partial	0.0833
ihb	Brainnetome	AROMA Non-Aggressive	GSR	glasso	0.0833
ihb	Brainnetome	aCompCor+24P	noGSR	glasso	0.0834
ihb	Schaefer200	AROMA Non-Aggressive	GSR	glasso	0.0835
ihb	Schaefer200	a/tCompCor(50%)+24P	GSR	glasso	0.0837
ihb	Brainnetome	24P	noGSR	glasso	0.0837
ihb	Brainnetome	AROMA Aggressive	GSR	glasso	0.0837
ihb	AAL	aCompCor(50%)+24P	noGSR	glasso	0.0838
ihb	Brainnetome	aCompCor+12P	noGSR	glasso	0.0838
ihb	Brainnetome	a/tCompCor(50%)+24P	GSR	glasso	0.0838
ihb	Schaefer200	aCompCor(50%)+24P	noGSR	glasso	0.084
ihb	Brainnetome	aCompCor(50%)+12P	GSR	glasso	0.084

ICC results

In Table S3 present top-20 pipelines, the table is ranked by masked ICC(3,1) score.

Table S3. Top-20 pipelines according to masked ICC(3,1)

Atlas	Strategy	GSR	FC	Mean ICC(3,1)	Mean ICC(2,1)	Mean ICC(1,1)
Schaefer200	24P	noGSR	tang	0.6712	0.6757	0.6769
Schaefer200	24P	GSR	tang	0.6613	0.6658	0.6672
Schaefer200	aCompCor+12P	noGSR	tang	0.6531	0.6577	0.6591
Schaefer200	AROMA Non-Aggressive	noGSR	tang	0.6525	0.6571	0.6585
Schaefer200	AROMA Non-Aggressive	GSR	tang	0.6465	0.6511	0.6526
Schaefer200	aCompCor+24P	noGSR	tang	0.644	0.6486	0.6501
Schaefer200	AROMA Aggressive	noGSR	tang	0.642	0.6466	0.6481
Schaefer200	aCompCor+12P	GSR	tang	0.6408	0.6455	0.647
Schaefer200	AROMA Aggressive	GSR	tang	0.6405	0.6451	0.6466
Schaefer200	aCompCor+24P	GSR	tang	0.6337	0.6384	0.64
Schaefer200	aCompCor(50%)+12P	noGSR	tang	0.6151	0.6199	0.6216
AAL	24P	noGSR	tang	0.6103	0.6151	0.6169
Brainnetome	24P	noGSR	tang	0.6088	0.6135	0.6153
Schaefer200	24P	noGSR	corr	0.6046	0.5919	0.5873
Schaefer200	aCompCor(50%)+24P	noGSR	tang	0.6038	0.6087	0.6105
Brainnetome	24P	noGSR	corr	0.6005	0.5915	0.5883
Schaefer200	aCompCor(50%)+12P	GSR	tang	0.5994	0.6042	0.6061
Brainnetome	AROMA Non-Aggressive	noGSR	tang	0.5983	0.603	0.6049
Brainnetome	AROMA Non-Aggressive	GSR	tang	0.5964	0.6011	0.603
AAL	24P	GSR	tang	0.5942	0.599	0.6009

Differences between masked and unmasked ICC

The difference in ICC scores between the masked and unmasked FC matrices is shown in Figures S1-S4 for every atlas.

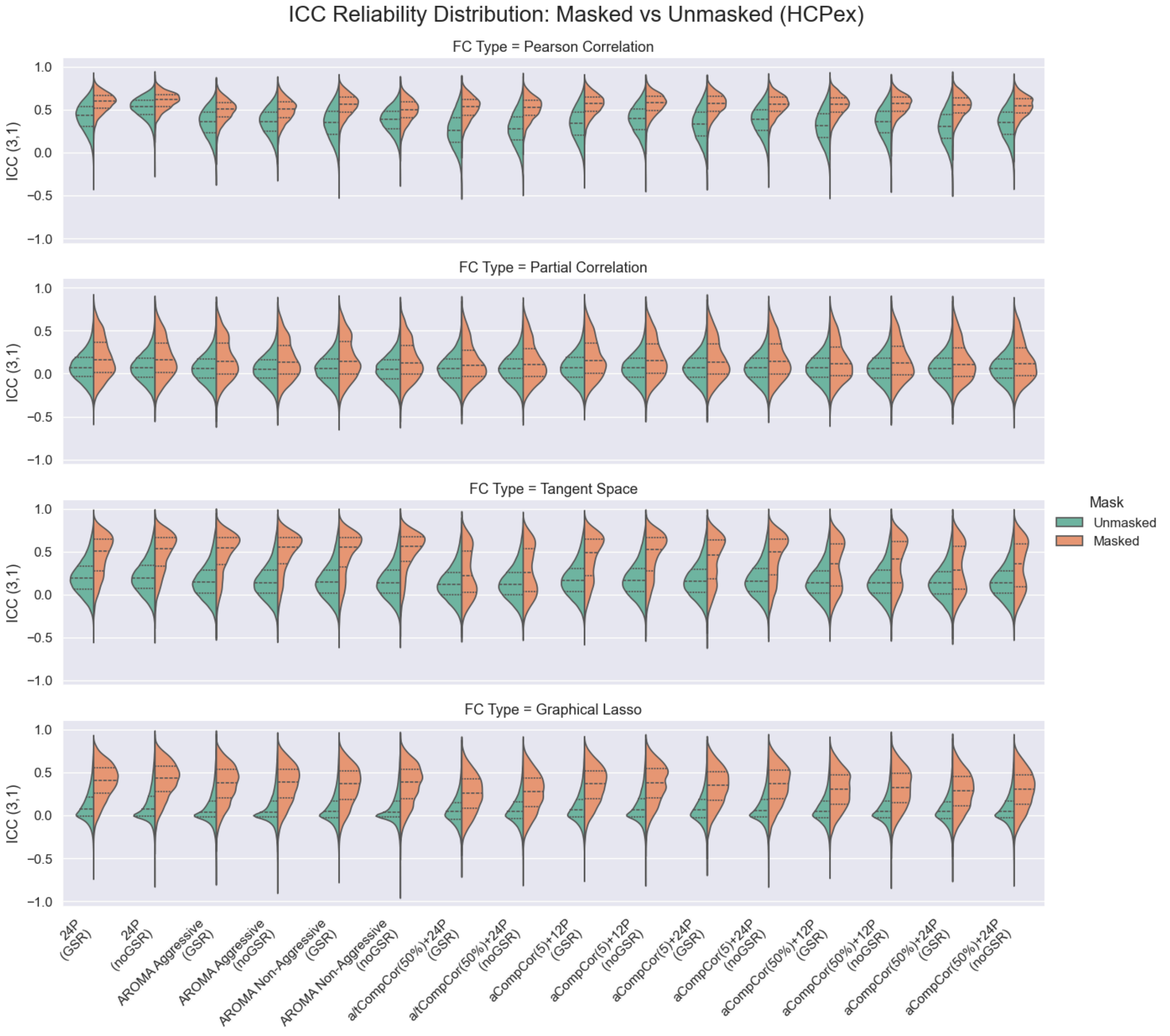

Figure S1

Comparison of ICC scores with (orange) and without masking (green) of near-zero FC weights. HCPex atlas. Every violin plot shows the distribution of ICC scores for every FC measure (rows) and strategies (columns). The central line within each violin plot represents the median. The upper and lower lines indicate the 25th percentile and the 75th percentile. There is no practical need in applying masking onto Pearson correlation, but we give it for comparison.

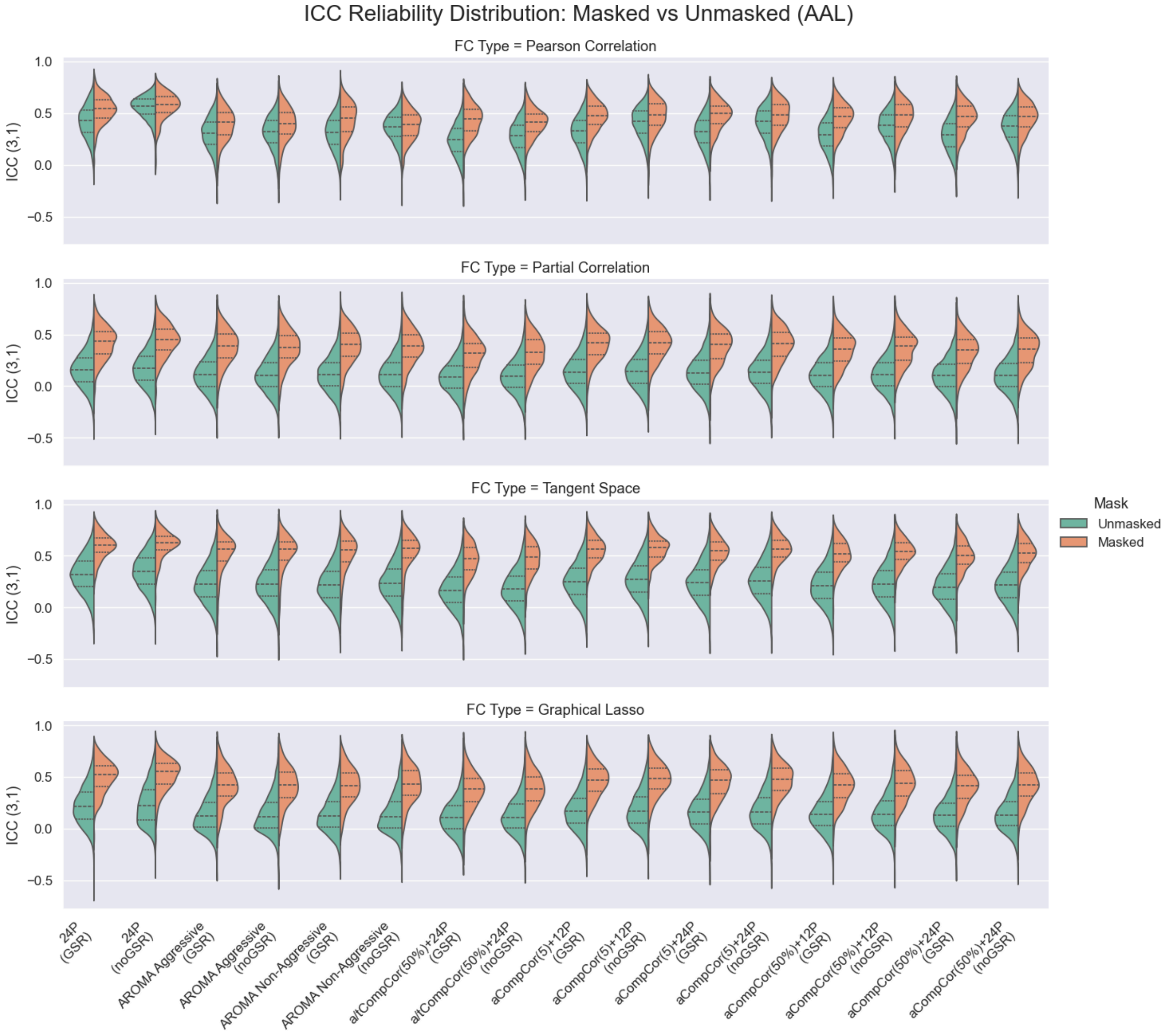

Figure S2

Comparison of ICC scores with (orange) and without masking (green) of near-zero FC weights. AAL atlas. Every violin plot shows the distribution of ICC scores for every FC measure (rows) and strategies (columns). The central line within each violin plot represents the median. The upper and lower lines indicate the 25th percentile and the 75th percentile. There is no practical need in applying masking onto Pearson correlation, but we give it for comparison.

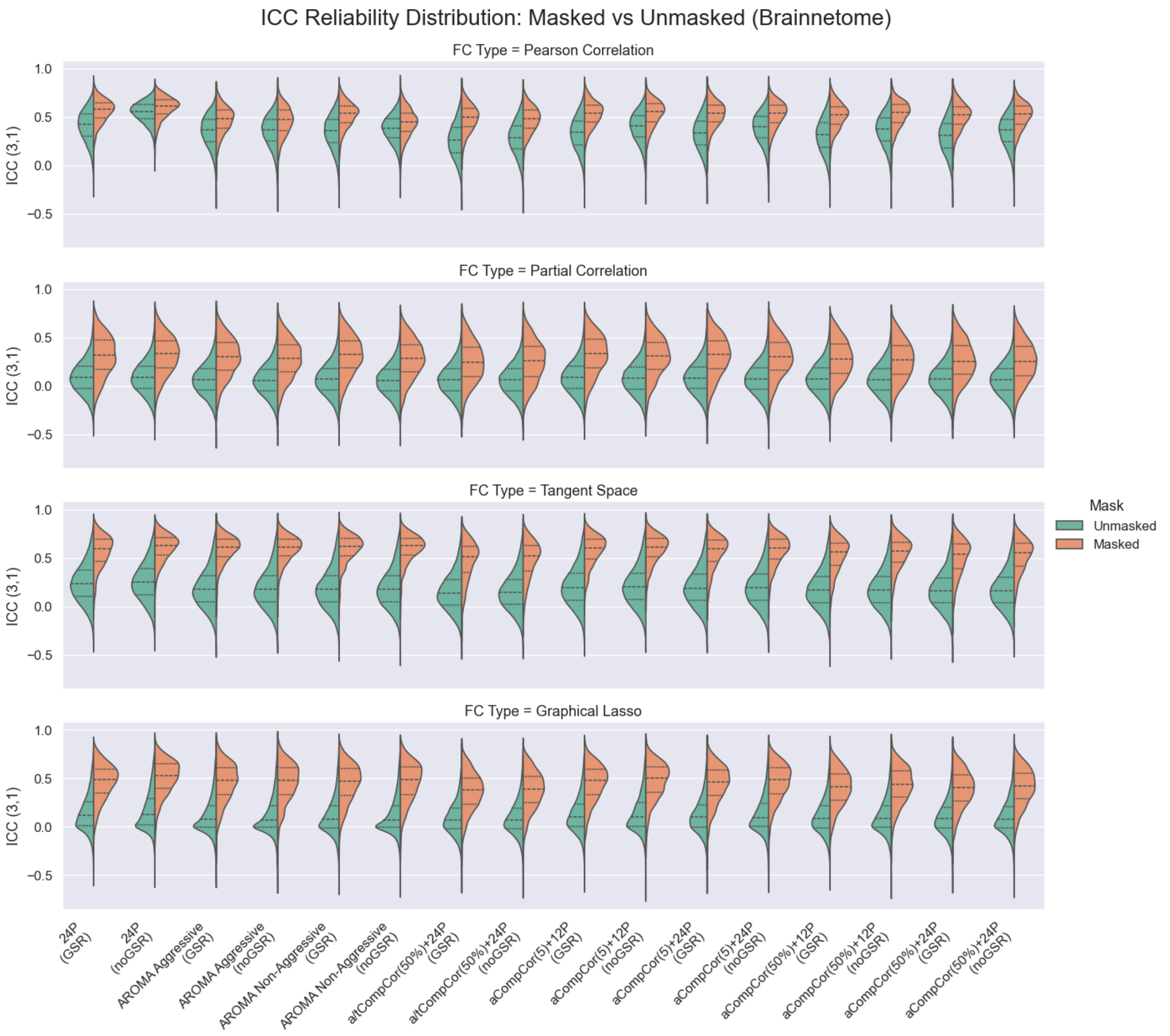

Figure S3

Comparison of ICC scores with (orange) and without masking (green) of near-zero FC weights. Brainnetome atlas. Every violin plot shows the distribution of ICC scores for every FC measure (rows) and strategies (columns). The central line within each violin plot represents the median. The upper and lower lines indicate the 25th percentile and the 75th percentile. There is no practical need in applying masking onto Pearson correlation, but we give it for comparison.

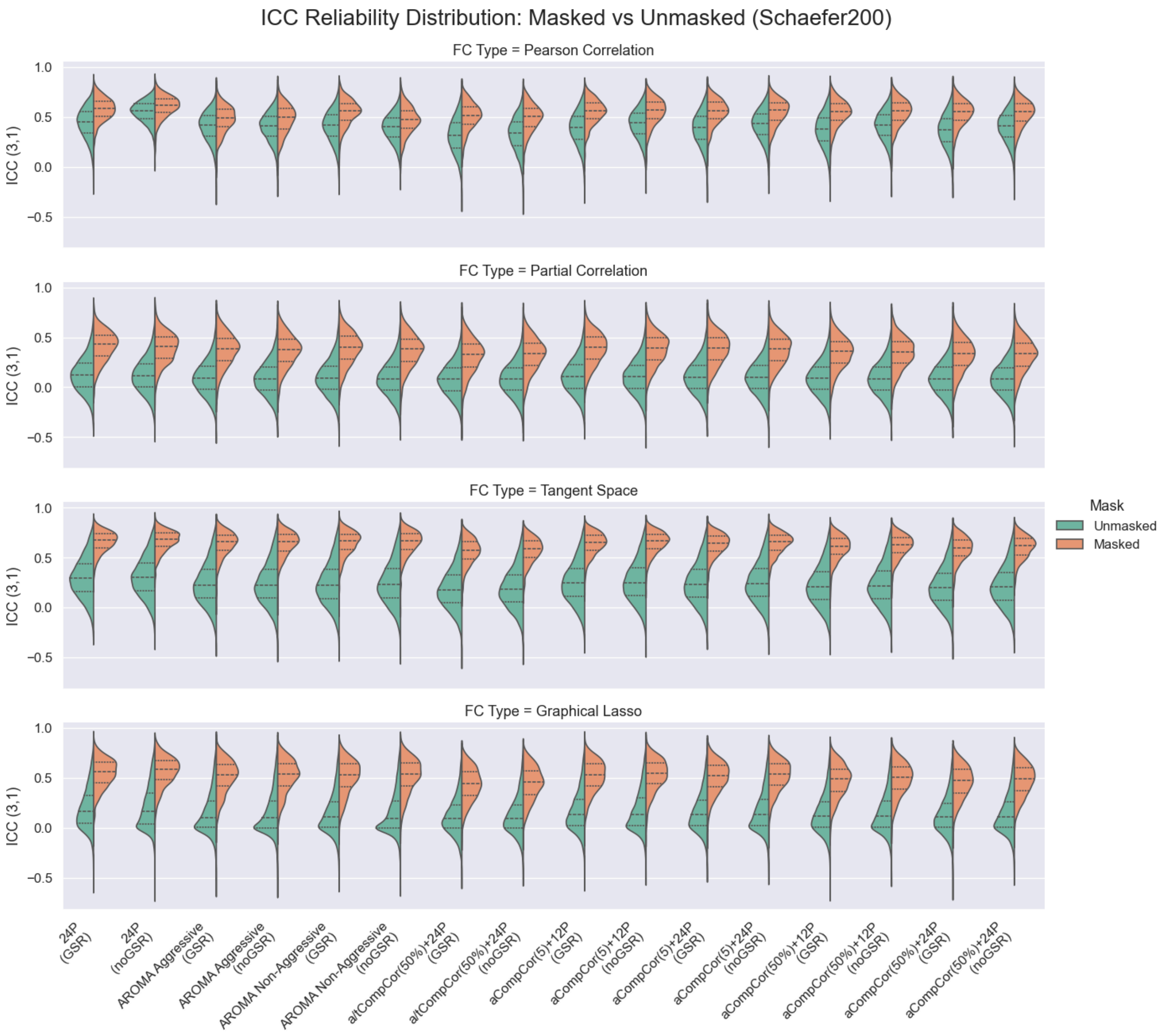

Figure S4

Comparison of ICC scores with (orange) and without masking (green) of near-zero FC weights. Schaefer200 atlas. Every violin plot shows the distribution of ICC scores for every FC measure (rows) and strategies (columns). The central line within each violin plot represents the median. The upper and lower lines indicate the 25th percentile and the 75th percentile. There is no practical need in applying masking onto Pearson correlation, but we give it for comparison.

Classification results

Table S4. Top 20 Pipelines (Mean Cross-Site Accuracy)

Atlas	Strategy	GSR	FC	China to IHB accuracy	IHB to China accuracy	Average cross-site accuracy
Brainnetome	aCompCor+12P	noGSR	tangent	0.8095	0.8646	0.8371
Brainnetome	aCompCor+24P	noGSR	tangent	0.7619	0.875	0.8185
AAL	aCompCor+12P	noGSR	tangent	0.7381	0.8958	0.817
AAL	aCompCor(50%)+24P	noGSR	tangent	0.7143	0.9062	0.8103
AAL	aCompCor+24P	noGSR	tangent	0.7024	0.9062	0.8043
Brainnetome	AROMA Aggressive	GSR	tangent	0.7738	0.8333	0.8036
AAL	aCompCor(50%)+12P	noGSR	tangent	0.7143	0.8854	0.7999
Schaefer200	aCompCor(50%)+12P	noGSR	tangent	0.7143	0.8854	0.7999
Schaefer200	aCompCor+12P	noGSR	tangent	0.7321	0.8646	0.7984
Schaefer200	aCompCor+24P	noGSR	tangent	0.7202	0.875	0.7976
Brainnetome	aCompCor+12P	GSR	corr	0.7083	0.8854	0.7969
Brainnetome	aCompCor(50%)+24P	noGSR	tangent	0.7321	0.8542	0.7932
Schaefer200	aCompCor(50%)+24P	noGSR	tangent	0.7262	0.8542	0.7902
Brainnetome	AROMA Aggressive	noGSR	tangent	0.744	0.8333	0.7887
AAL	aCompCor+12P	GSR	tangent	0.7202	0.8542	0.7872
AAL	AROMA Non-Aggressive	GSR	tangent	0.6667	0.9062	0.7865
AAL	aCompCor(50%)+12P	GSR	tangent	0.6845	0.8854	0.785
AAL	aCompCor+24P	GSR	tangent	0.7024	0.8646	0.7835
HCPex	aCompCor+12P	noGSR	corr	0.6905	0.875	0.7827
HCPex	aCompCor(50%)+12P	noGSR	corr	0.6786	0.8854	0.782

Table S5. Top 20 Pipelines (Mean Cross-Site ROC-AUC)

Atlas	Strategy	GSR	FC	China to IHB ROC-AUC	IHB to China ROC-AUC	Average cross-site ROC-AUC
Brainnetome	24P	GSR	tangent	0.9082	0.9362	0.9222
Schaefer200	AROMA Non-Aggressive	GSR	tangent	0.8733	0.9553	0.9143
Schaefer200	aCompCor+12P	noGSR	tangent	0.8839	0.9392	0.9116
HCPex	aCompCor+12P	noGSR	tangent	0.8865	0.9362	0.9113
HCPex	24P	GSR	tangent	0.8802	0.9401	0.9102
Brainnetome	24P	noGSR	tangent	0.8921	0.9266	0.9094
HCPex	aCompCor+24P	noGSR	tangent	0.8831	0.9332	0.9081
Schaefer200	aCompCor(50%)+12P	noGSR	tangent	0.8678	0.9457	0.9068
Schaefer200	aCompCor+24P	noGSR	tangent	0.8764	0.9371	0.9067
Brainnetome	AROMA Non-Aggressive	GSR	tangent	0.87	0.9423	0.9062
Brainnetome	aCompCor+12P	noGSR	tangent	0.8829	0.9275	0.9052
Brainnetome	aCompCor+12P	GSR	tangent	0.8754	0.9349	0.9052
Schaefer200	AROMA Non-Aggressive	noGSR	tangent	0.87	0.9401	0.9051
Schaefer200	aCompCor(50%)+12P	GSR	tangent	0.873	0.9366	0.9048
Schaefer200	aCompCor(50%)+24P	noGSR	tangent	0.8669	0.9427	0.9048
Brainnetome	aCompCor+24P	noGSR	tangent	0.8729	0.9362	0.9045
Brainnetome	aCompCor+24P	GSR	tangent	0.8712	0.9375	0.9043
HCPex	24P	noGSR	tangent	0.8581	0.9457	0.9019
Brainnetome	aCompCor(50%)+24P	GSR	tangent	0.8668	0.9358	0.9013

HCPex	aCompCor+12P	GSR	tangent	0.8601	0.9405	0.9003

Similarity between denoising strategies

China dataset

AAL

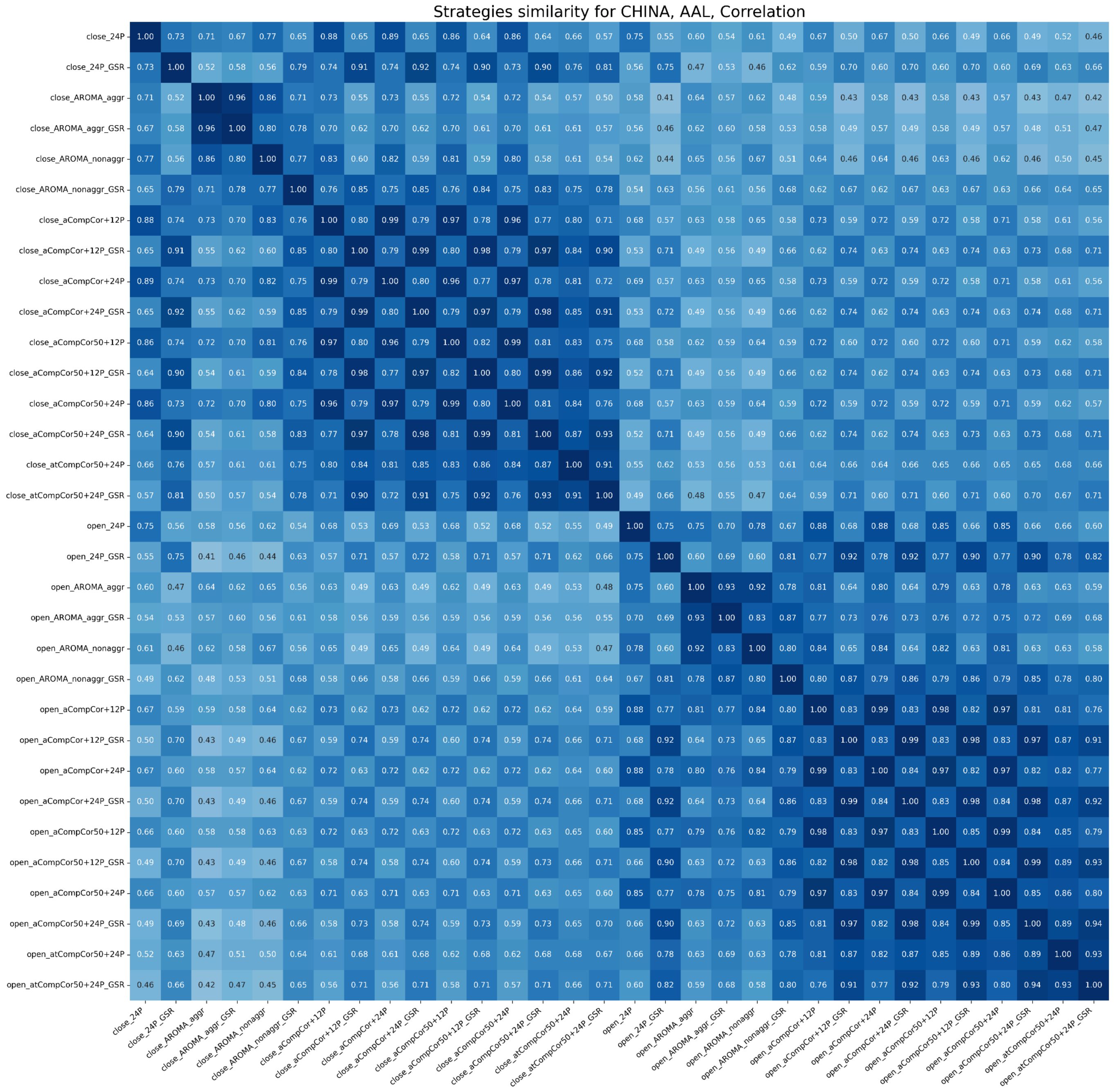

Figure S5.

Similarity between signals of both EO and EC states denoised using different strategies for the China dataset, the AAL atlas, correlation FC. The heatmap shows similarity for one FC measure. The color range is from white (0, low similarity) to dark blue (1, high similarity). Mean values across subjects are presented.

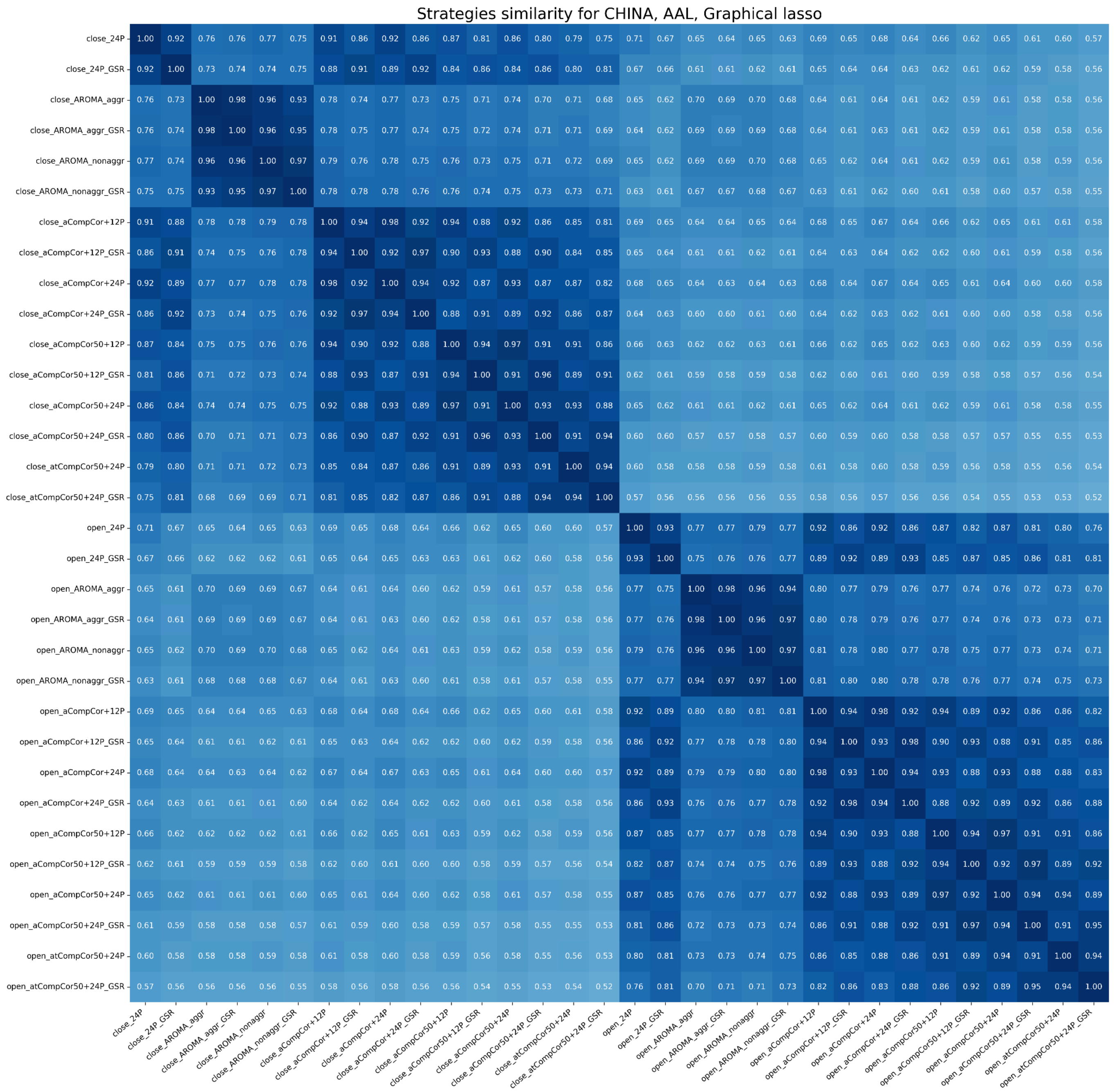

Figure S6.

Similarity between signals of both EO and EC states denoised using different strategies for the China dataset, the AAL atlas, glasso FC. The heatmap shows similarity for one FC measure. The color range is from white (0, low similarity) to dark blue (1, high similarity). Mean values across subjects are presented.

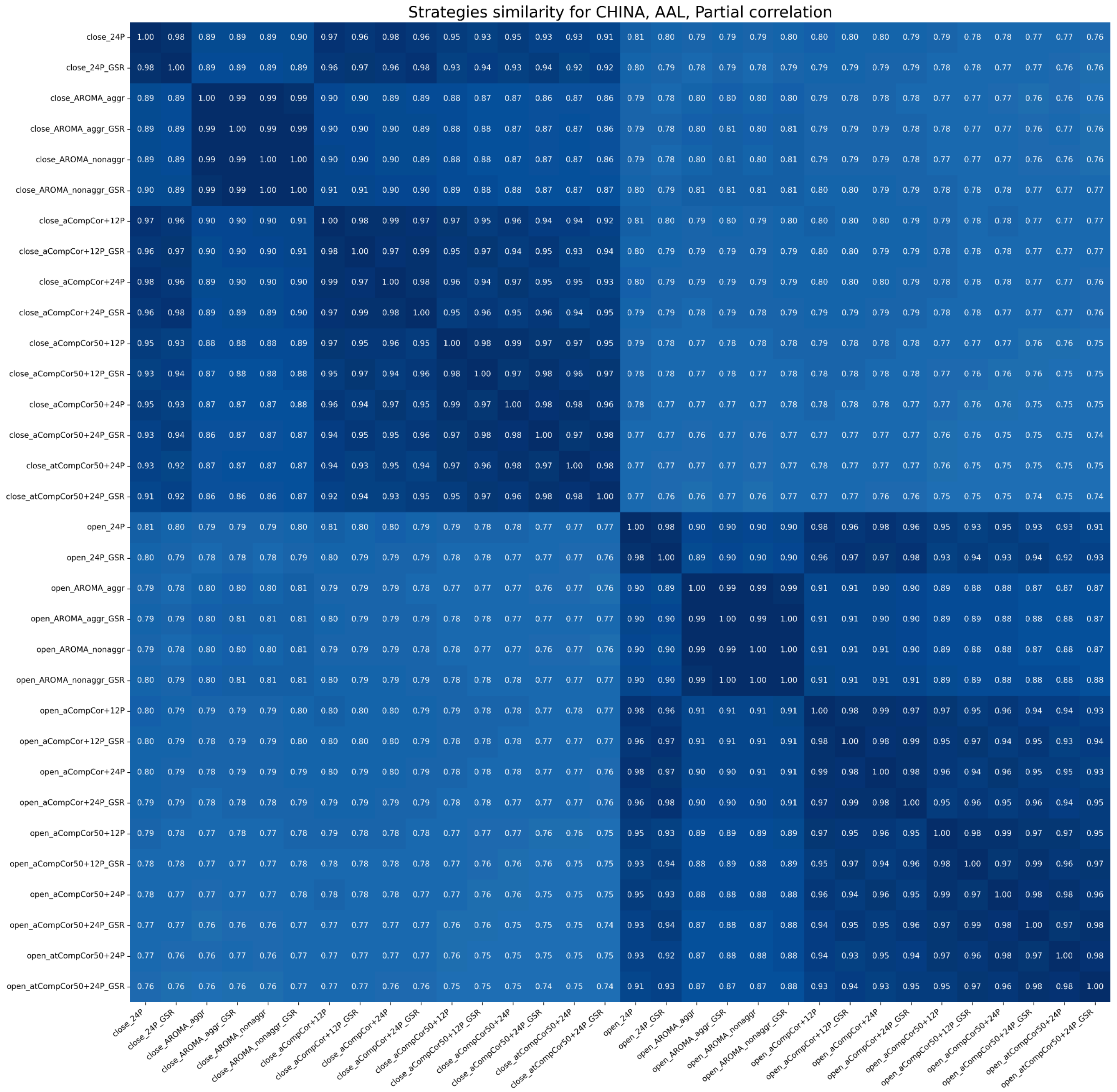

Figure S7.

Similarity between signals of both EO and EC states denoised using different strategies for the China dataset, the AAL atlas, partial correlation FC. The heatmap shows similarity for one FC measure. The color range is from white (0, low similarity) to dark blue (1, high similarity). Mean values across subjects are presented.

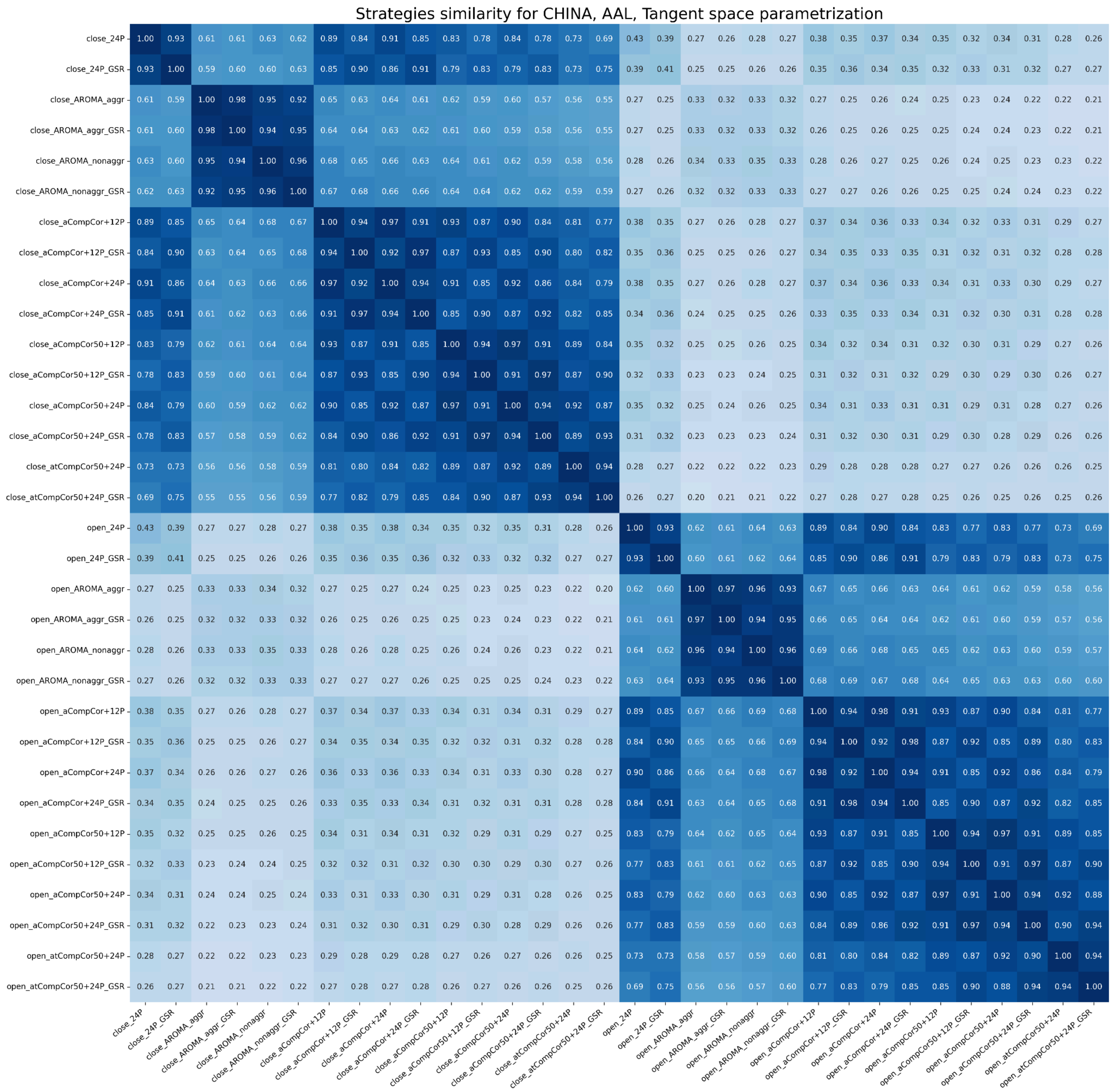

Figure S8.

Similarity between signals of both EO and EC states denoised using different strategies for the China dataset, the AAL atlas, tangent space FC. The heatmap shows similarity for one FC measure. The color range is from white (0, low similarity) to dark blue (1, high similarity). Mean values across subjects are presented.

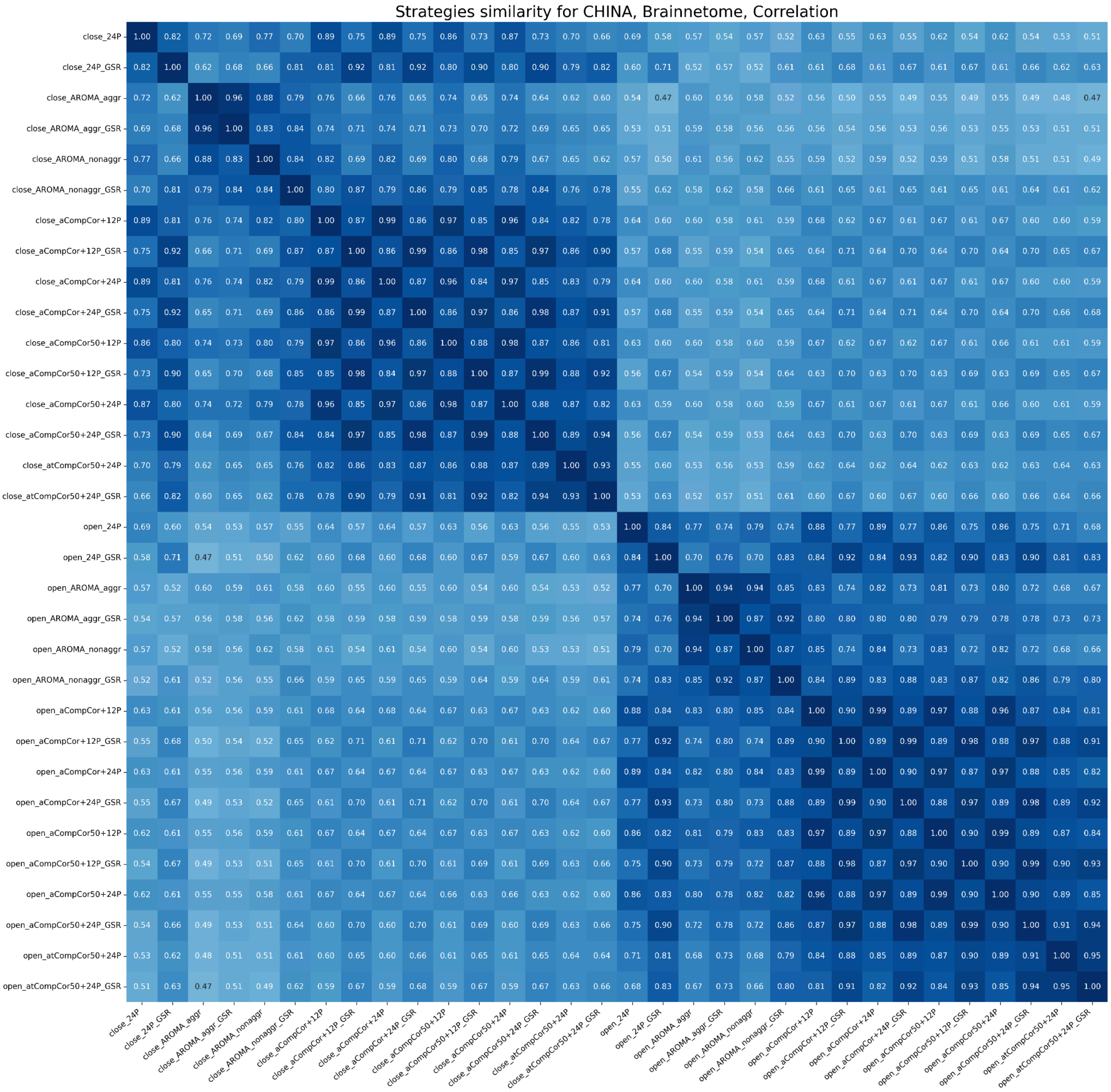

Figure S9.

Similarity between signals of both EO and EC states denoised using different strategies for the China dataset, the Brainnetome atlas, correlation FC. The heatmap shows similarity for one FC measure. The color range is from white (0, low similarity) to dark blue (1, high similarity). Mean values across subjects are presented.

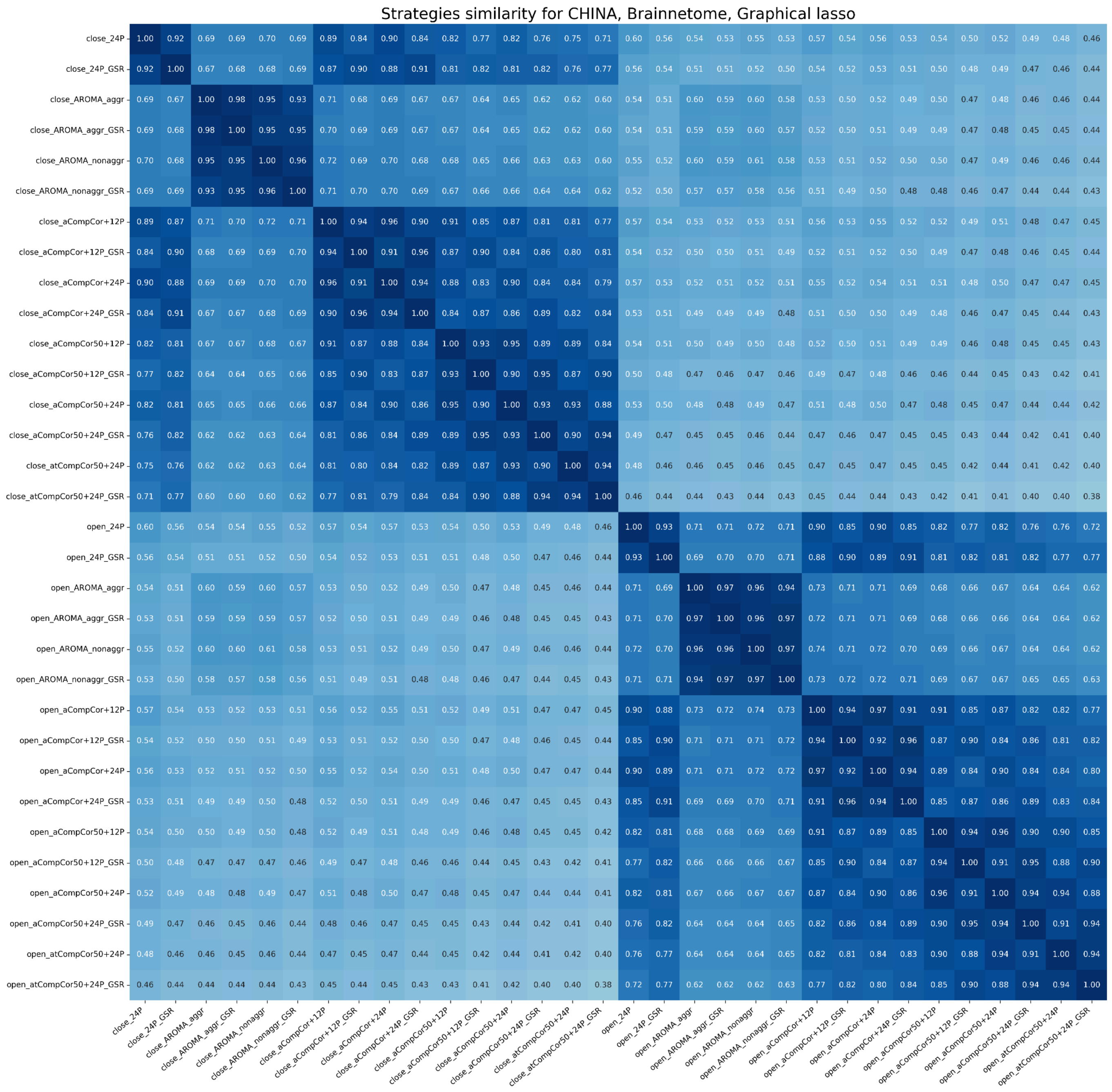

Figure S10.

Similarity between signals of both EO and EC states denoised using different strategies for the China dataset, the Brainnetome atlas, glasso FC. The heatmap shows similarity for one FC measure. The color range is from white (0, low similarity) to dark blue (1, high similarity). Mean values across subjects are presented.

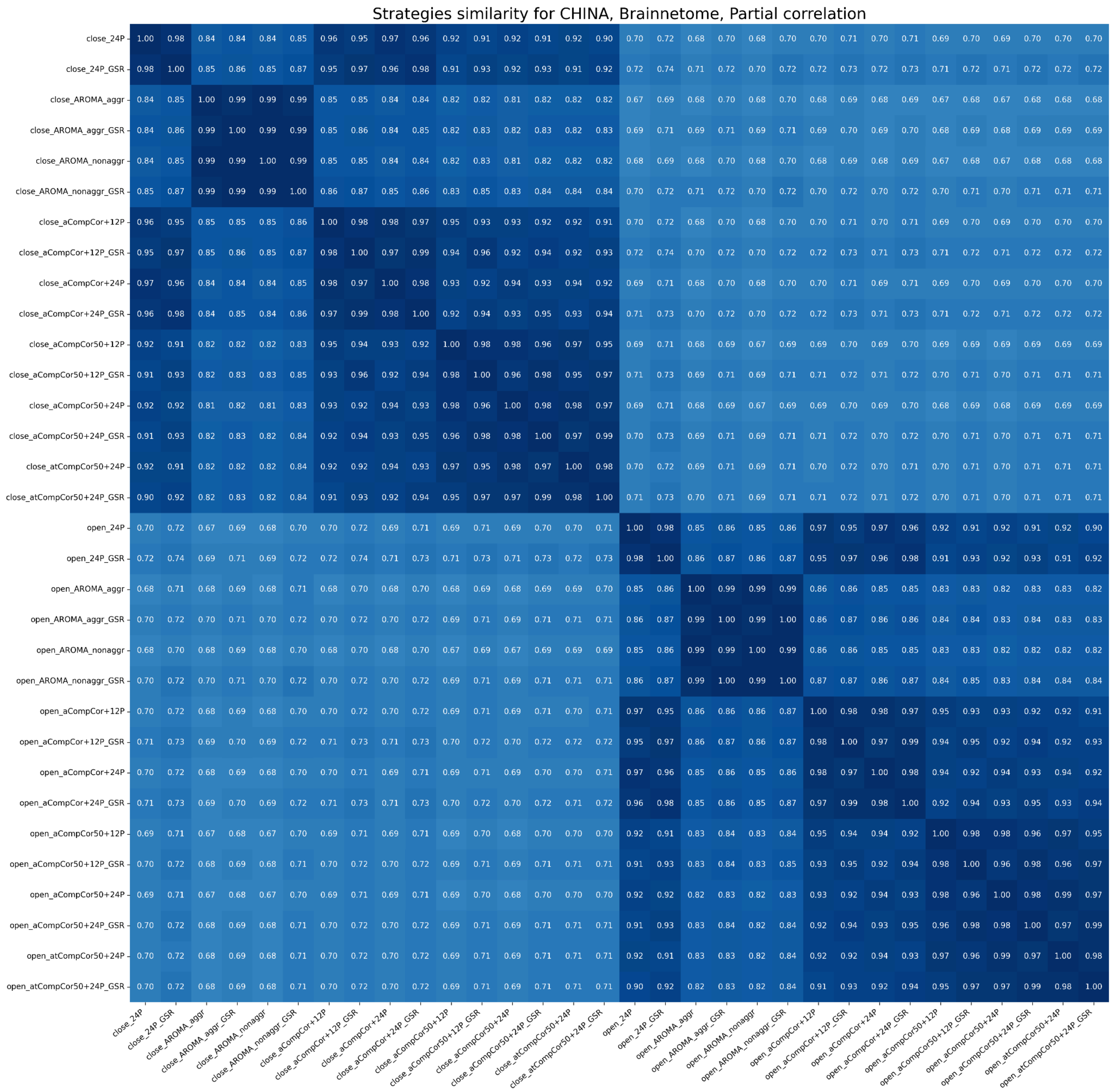

Figure S11.

Similarity between signals of both EO and EC states denoised using different strategies for the China dataset, the Brainnetome atlas, partial correlation FC. The heatmap shows similarity for one FC measure. The color range is from white (0, low similarity) to dark blue (1, high similarity). Mean values across subjects are presented.

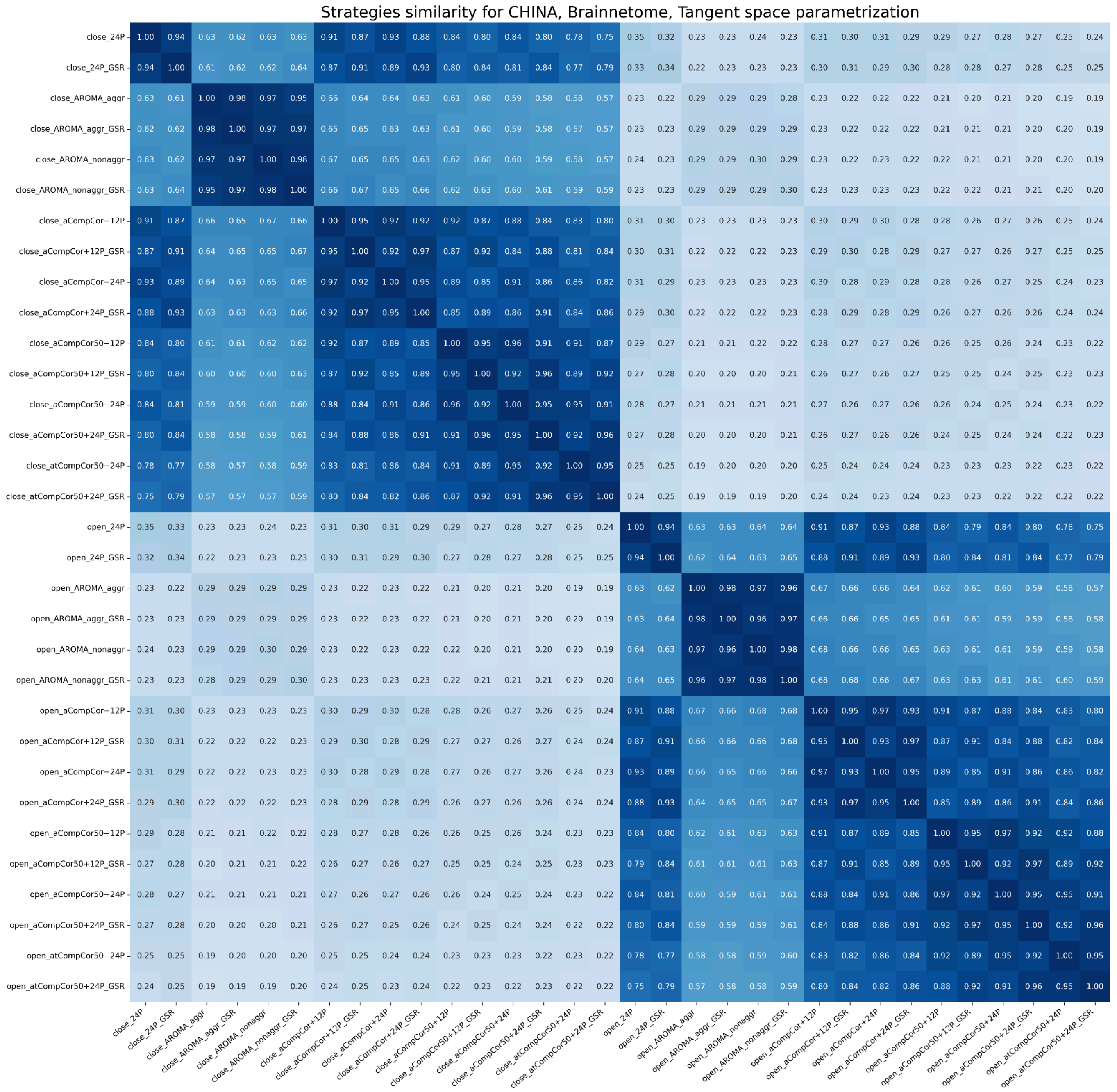

Figure S12.

Similarity between signals of both EO and EC states denoised using different strategies for the China dataset, the Brainnetome atlas, tangent space FC. The heatmap shows similarity for one FC measure. The color range is from white (0, low similarity) to dark blue (1, high similarity). Mean values across subjects are presented.

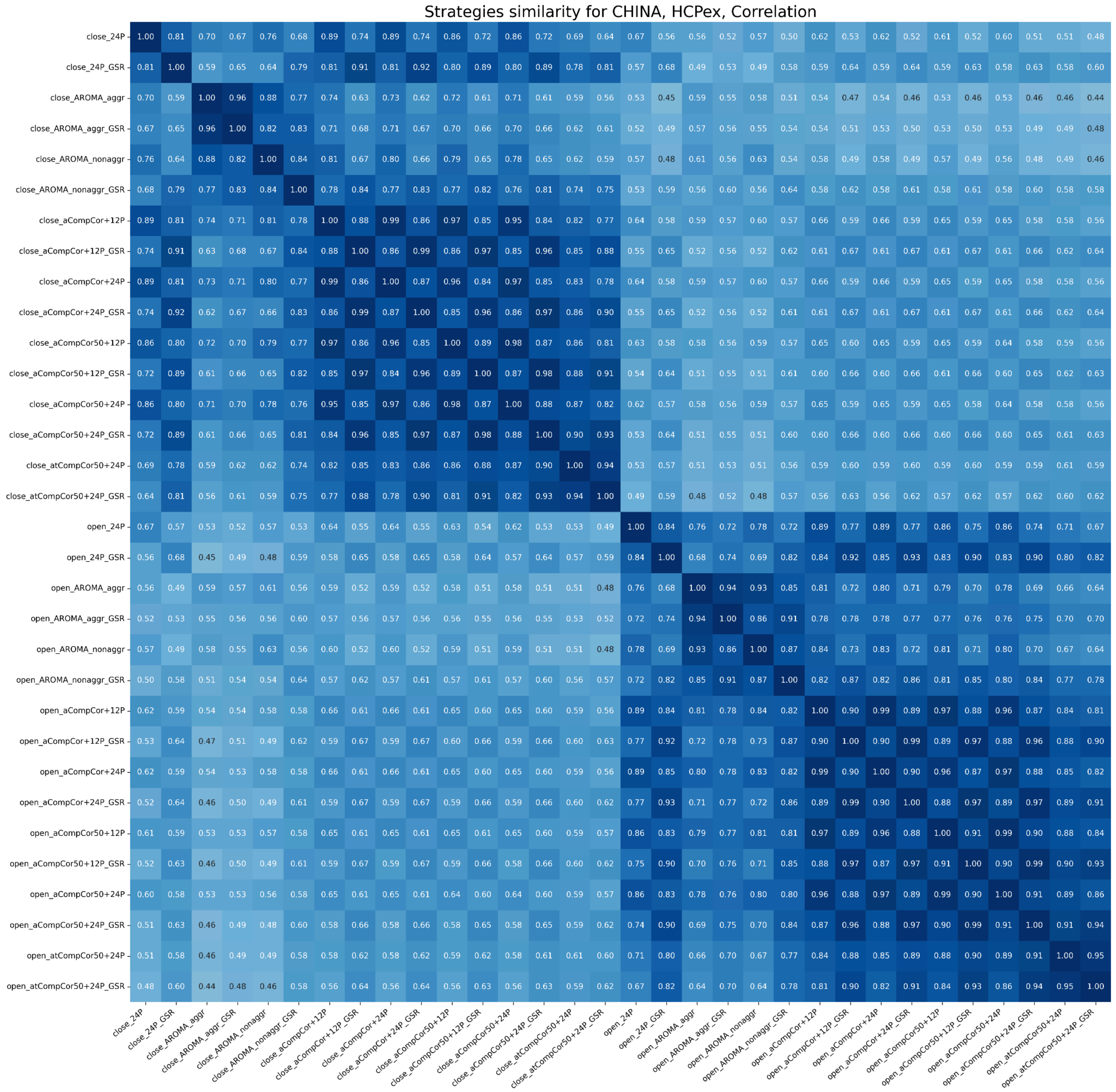

Figure S13.

Similarity between signals of both EO and EC states denoised using different strategies for the China dataset, the HCPex atlas, correlation FC. The heatmap shows similarity for one FC measure. The color range is from white (0, low similarity) to dark blue (1, high similarity). Mean values across subjects are presented.

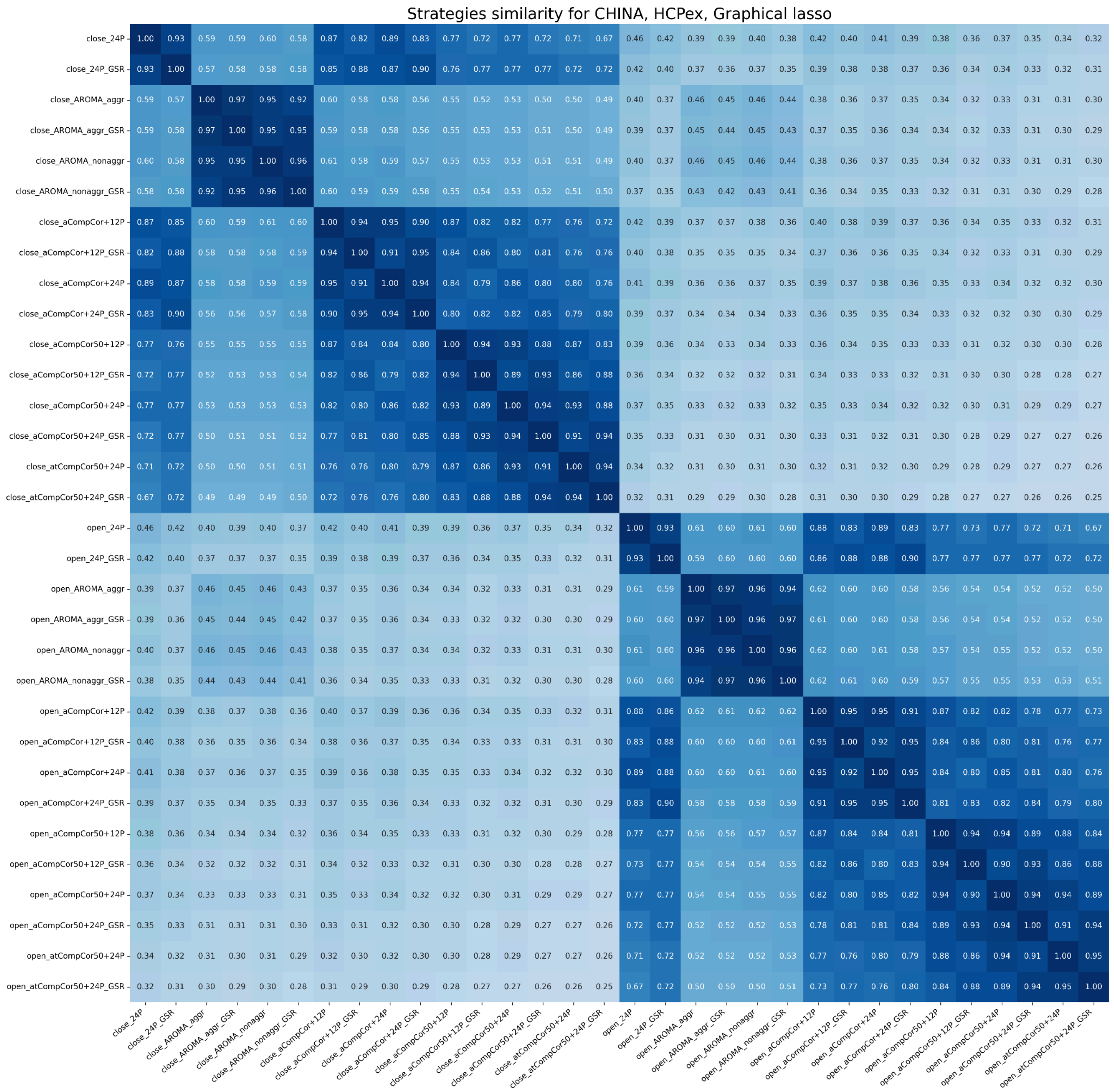

Figure S14.

Similarity between signals of both EO and EC states denoised using different strategies for the China dataset, the HCPex atlas, correlation FC. The heatmap shows similarity for one FC measure. The color range is from white (0, low similarity) to dark blue (1, high similarity). Mean values across subjects are presented.

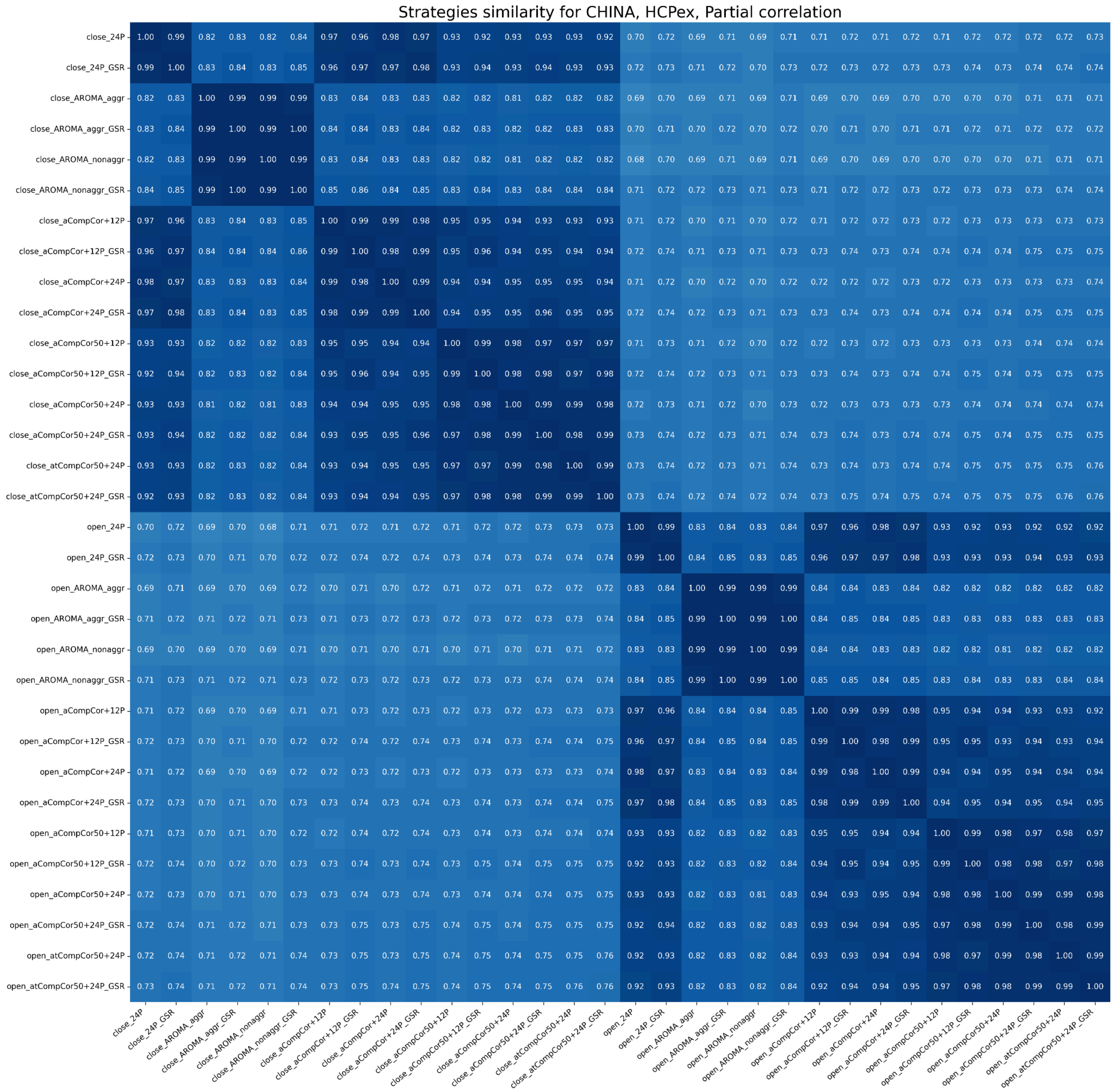

Figure S15.

Similarity between signals of both EO and EC states denoised using different strategies for the China dataset, the HCPex atlas, partial correlation FC. The heatmap shows similarity for one FC measure. The color range is from white (0, low similarity) to dark blue (1, high similarity). Mean values across subjects are presented.

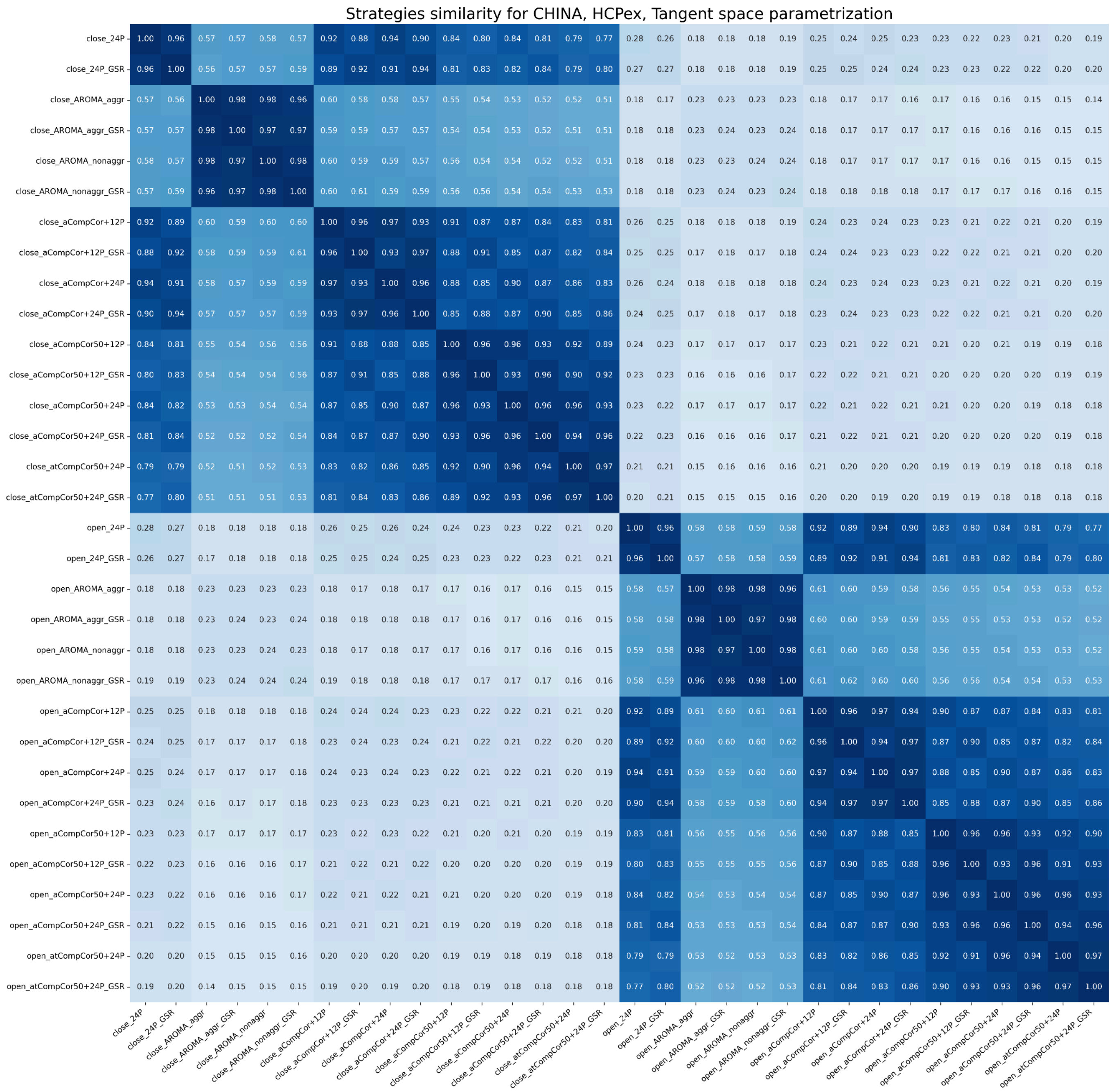

Figure S16.

Similarity between signals of both EO and EC states denoised using different strategies for the China dataset, the HCPex atlas, tangent space FC. The heatmap shows similarity for one FC measure. The color range is from white (0, low similarity) to dark blue (1, high similarity). Mean values across subjects are presented.

Schaefer200

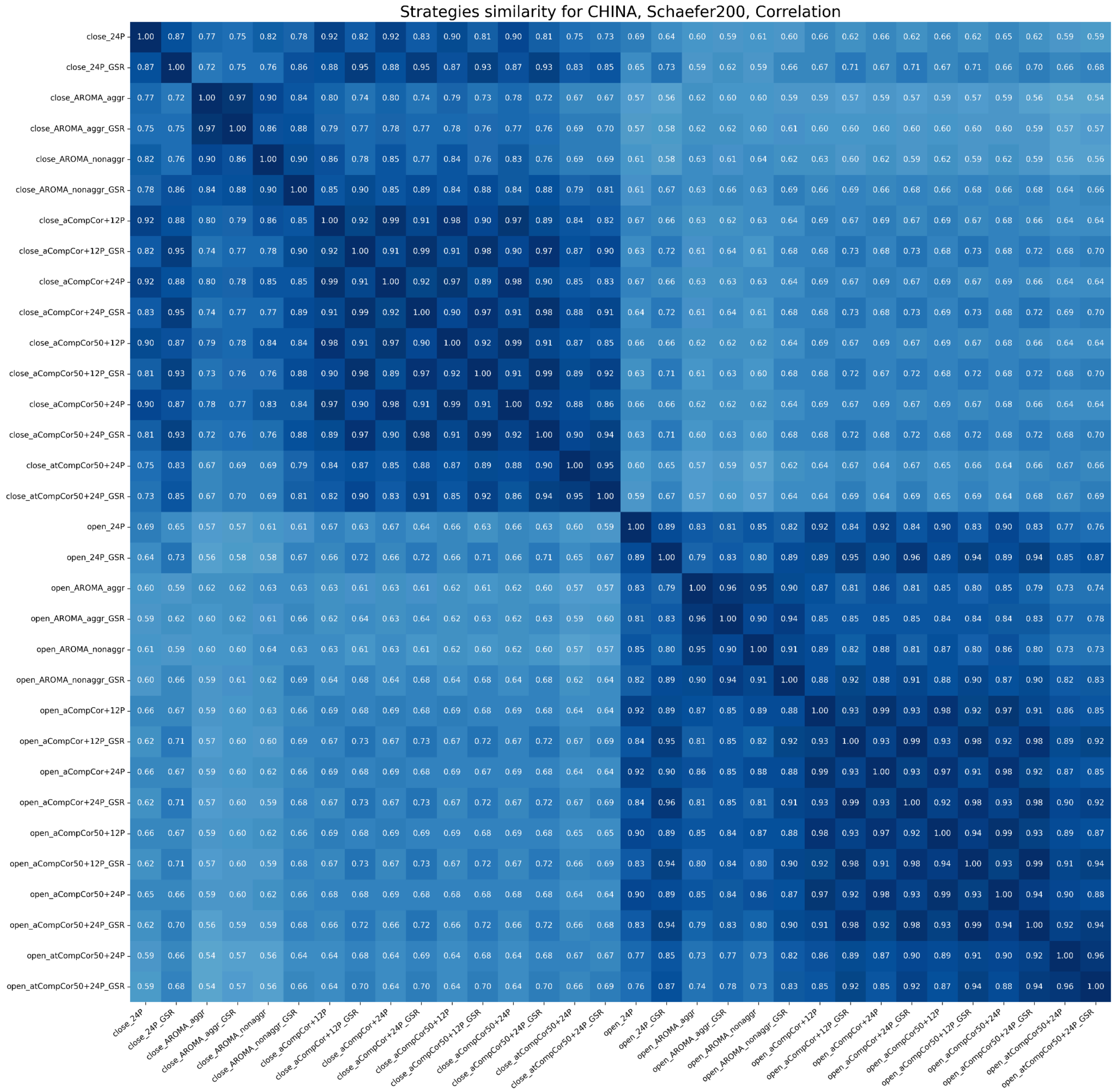

Figure S17.

Similarity between signals of both EO and EC states denoised using different strategies for the China dataset, the Schaefer200 atlas, correlation FC. The heatmap shows similarity for one FC measure. The color range is from white (0, low similarity) to dark blue (1, high similarity). Mean values across subjects are presented.

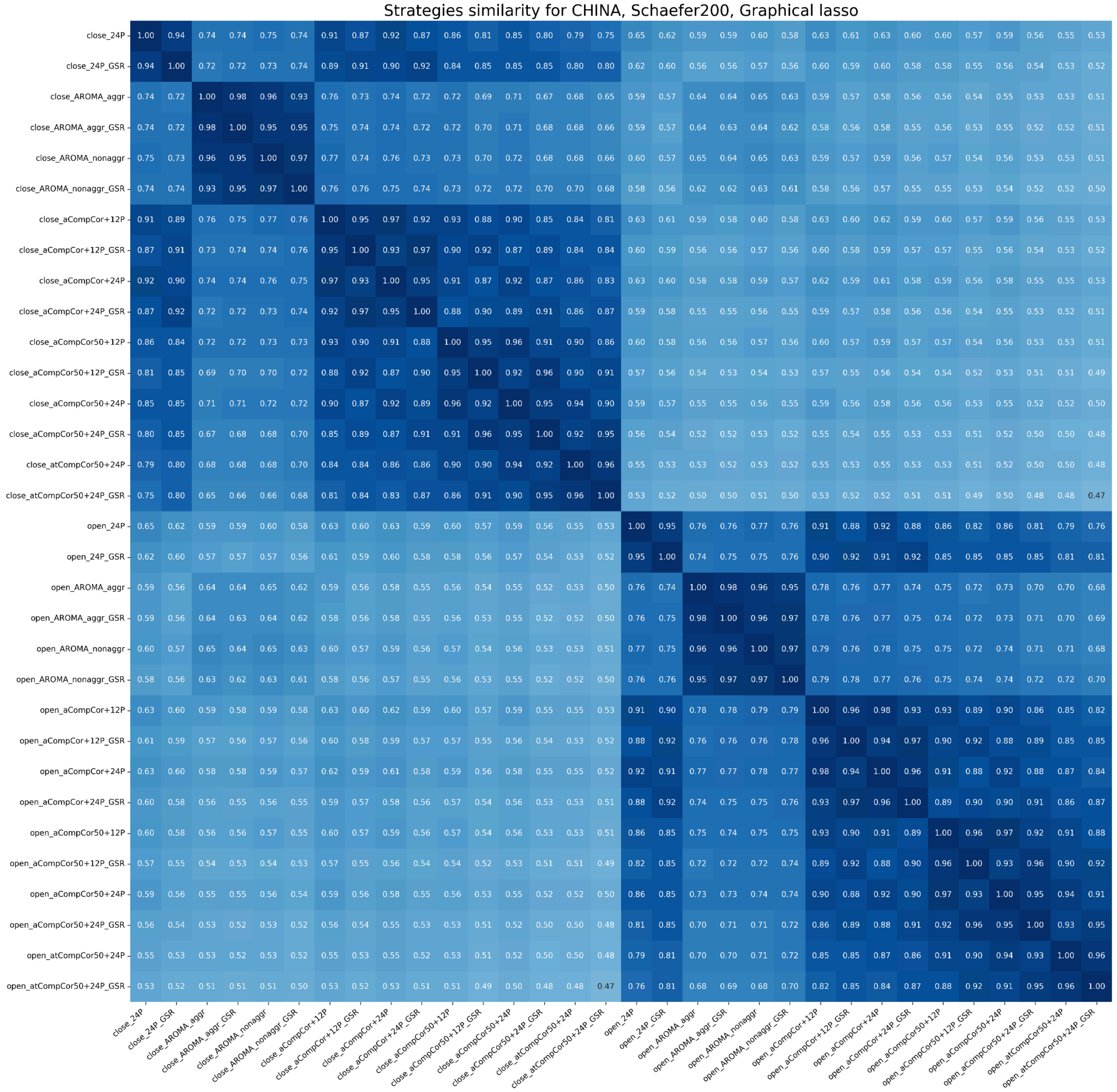

Figure S18.

Similarity between signals of both EO and EC states denoised using different strategies for the China dataset, the Schaefer200 atlas, glasso FC. The heatmap shows similarity for one FC measure. The color range is from white (0, low similarity) to dark blue (1, high similarity). Mean values across subjects are presented.

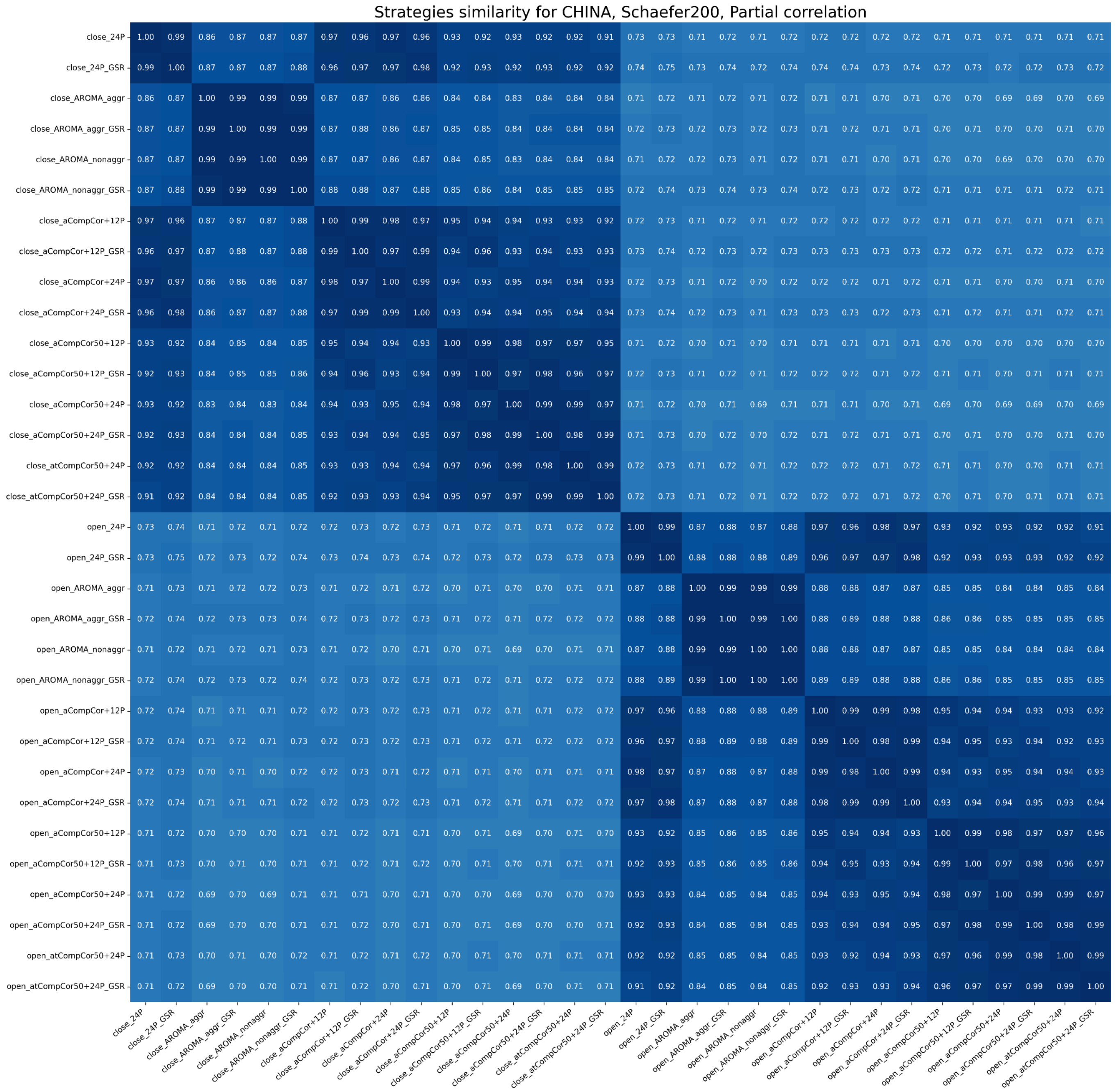

Figure S19.

Similarity between signals of both EO and EC states denoised using different strategies for the China dataset, the Schaefer200 atlas, partial correlation FC. The heatmap shows similarity for one FC measure. The color range is from white (0, low similarity) to dark blue (1, high similarity). Mean values across subjects are presented.

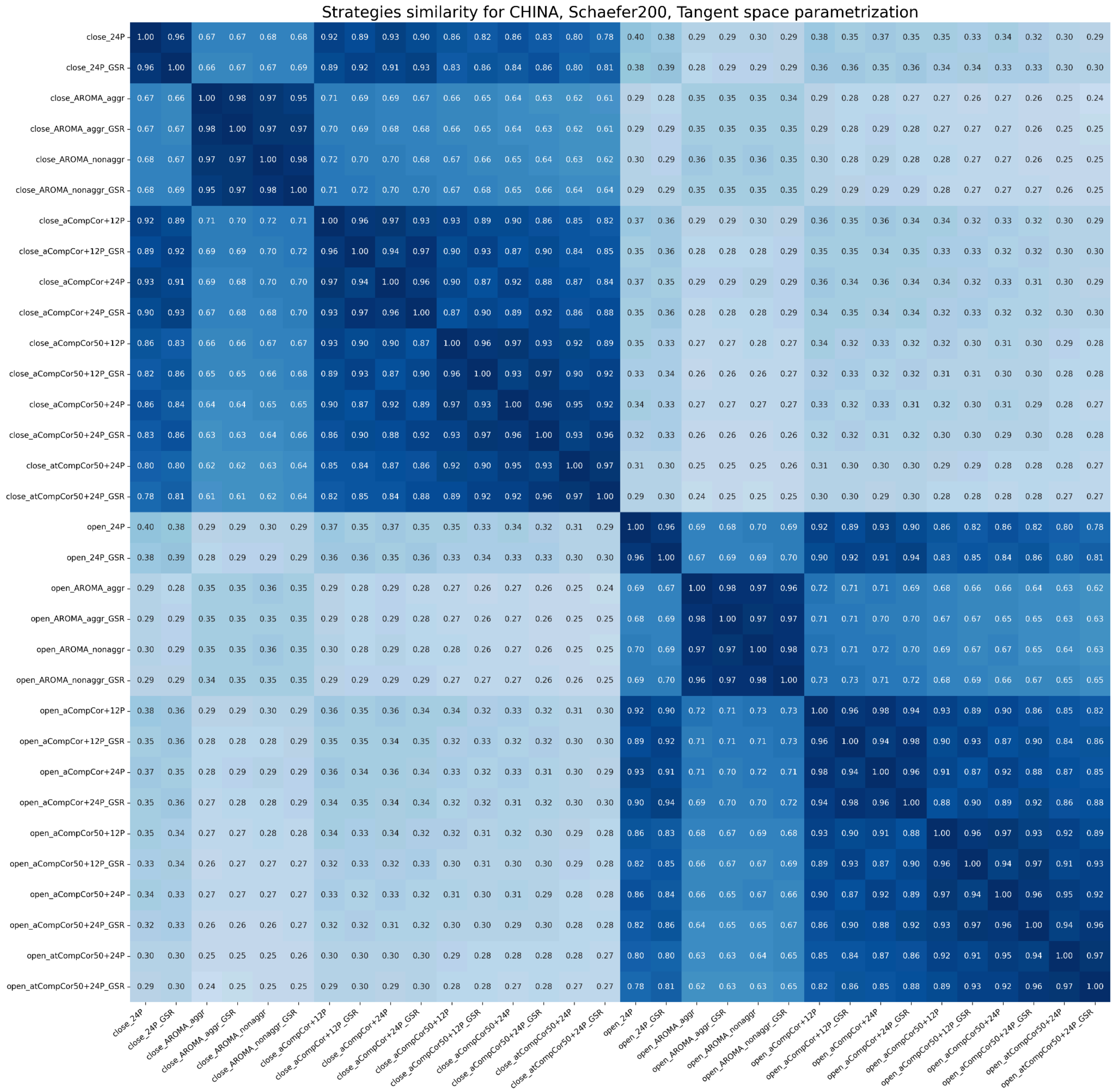

Figure S20.

Similarity between signals of both EO and EC states denoised using different strategies for the China dataset, the Schaefer200 atlas, tangent space FC. The heatmap shows similarity for one FC measure. The color range is from white (0, low similarity) to dark blue (1, high similarity). Mean values across subjects are presented.

IHB dataset

AAL

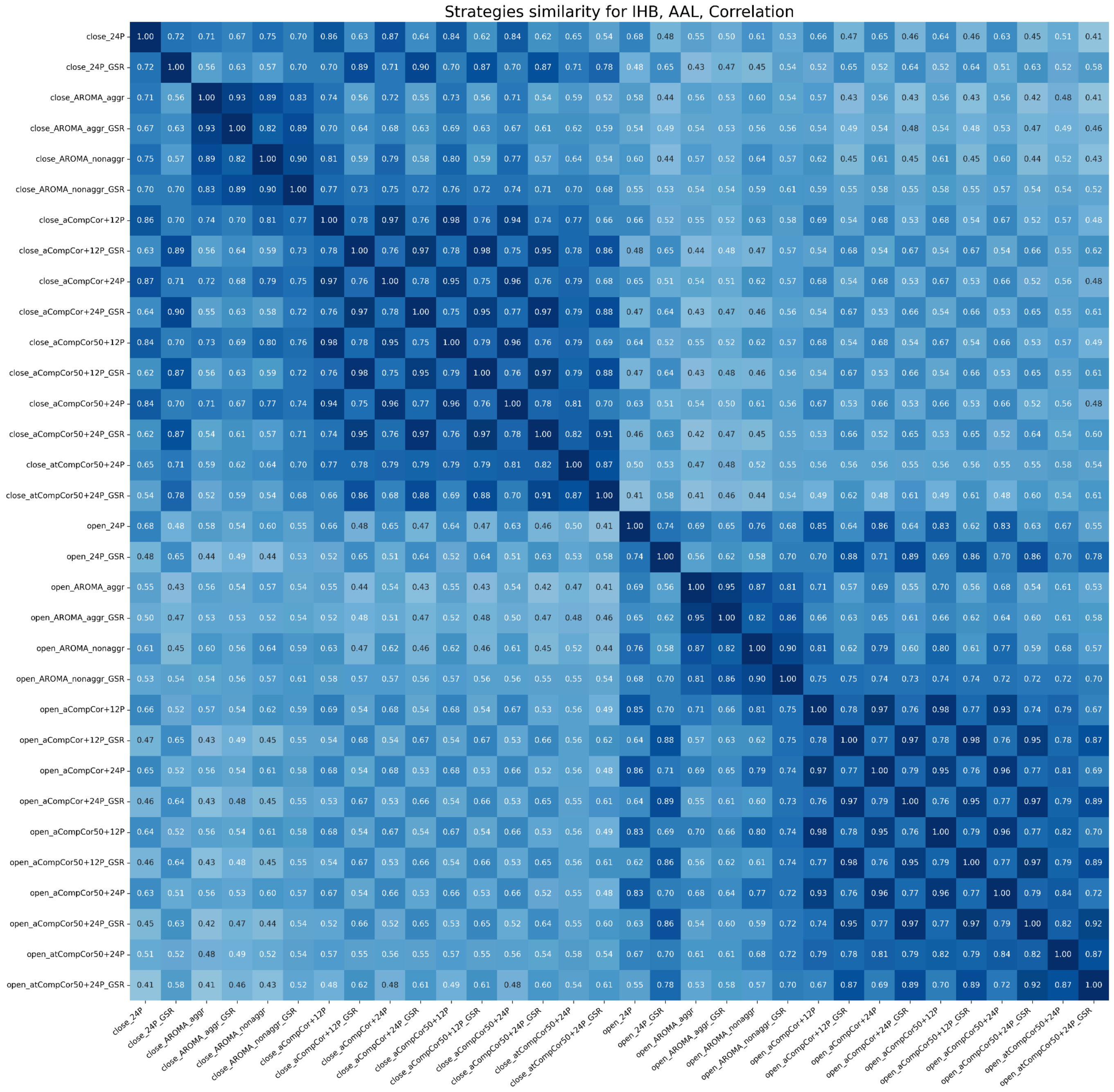

Figure S21.

Similarity between signals of both EO and EC states denoised using different strategies for the IHB dataset, the AAL atlas, correlation FC. The heatmap shows similarity for one FC measure. The color range is from white (0, low similarity) to dark blue (1, high similarity). Mean values across subjects are presented.

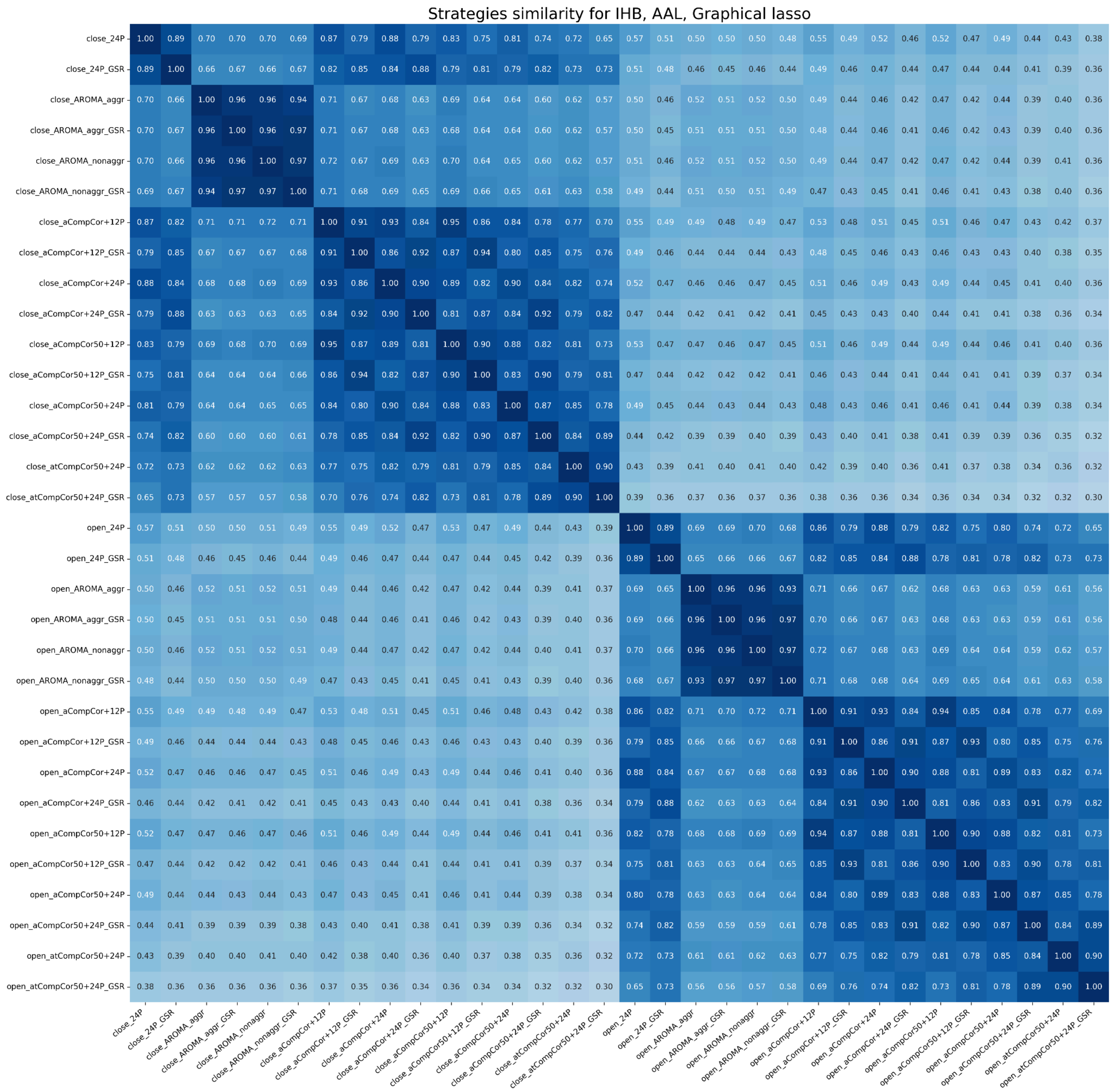

Figure S22.

Similarity between signals of both EO and EC states denoised using different strategies for the IHB dataset, the AAL atlas, glasso FC. The heatmap shows similarity for one FC measure. The color range is from white (0, low similarity) to dark blue (1, high similarity). Mean values across subjects are presented.

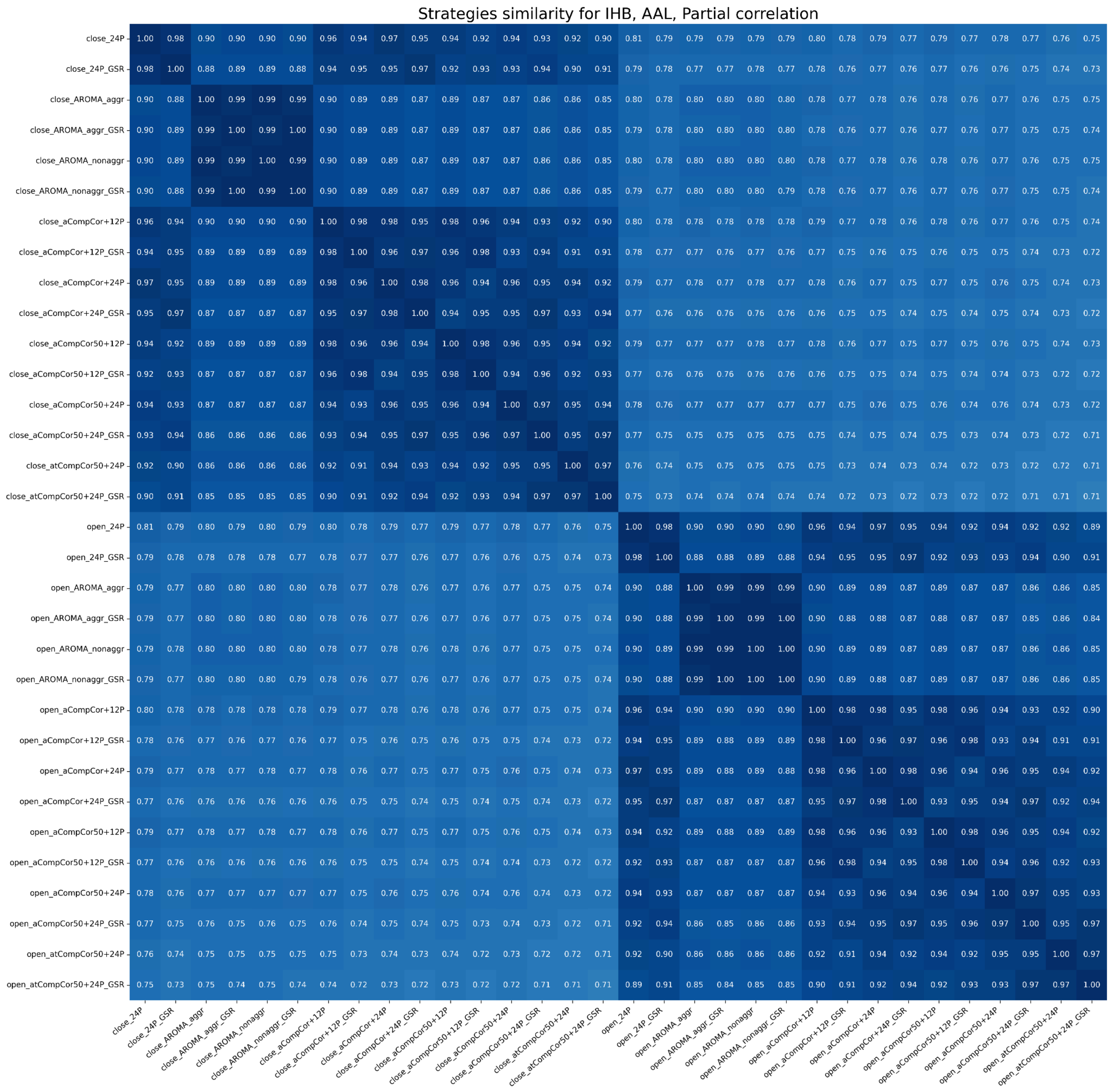

Figure S23.

Similarity between signals of both EO and EC states denoised using different strategies for the IHB dataset, the AAL atlas, partial correlation FC. The heatmap shows similarity for one FC measure. The color range is from white (0, low similarity) to dark blue (1, high similarity). Mean values across subjects are presented.

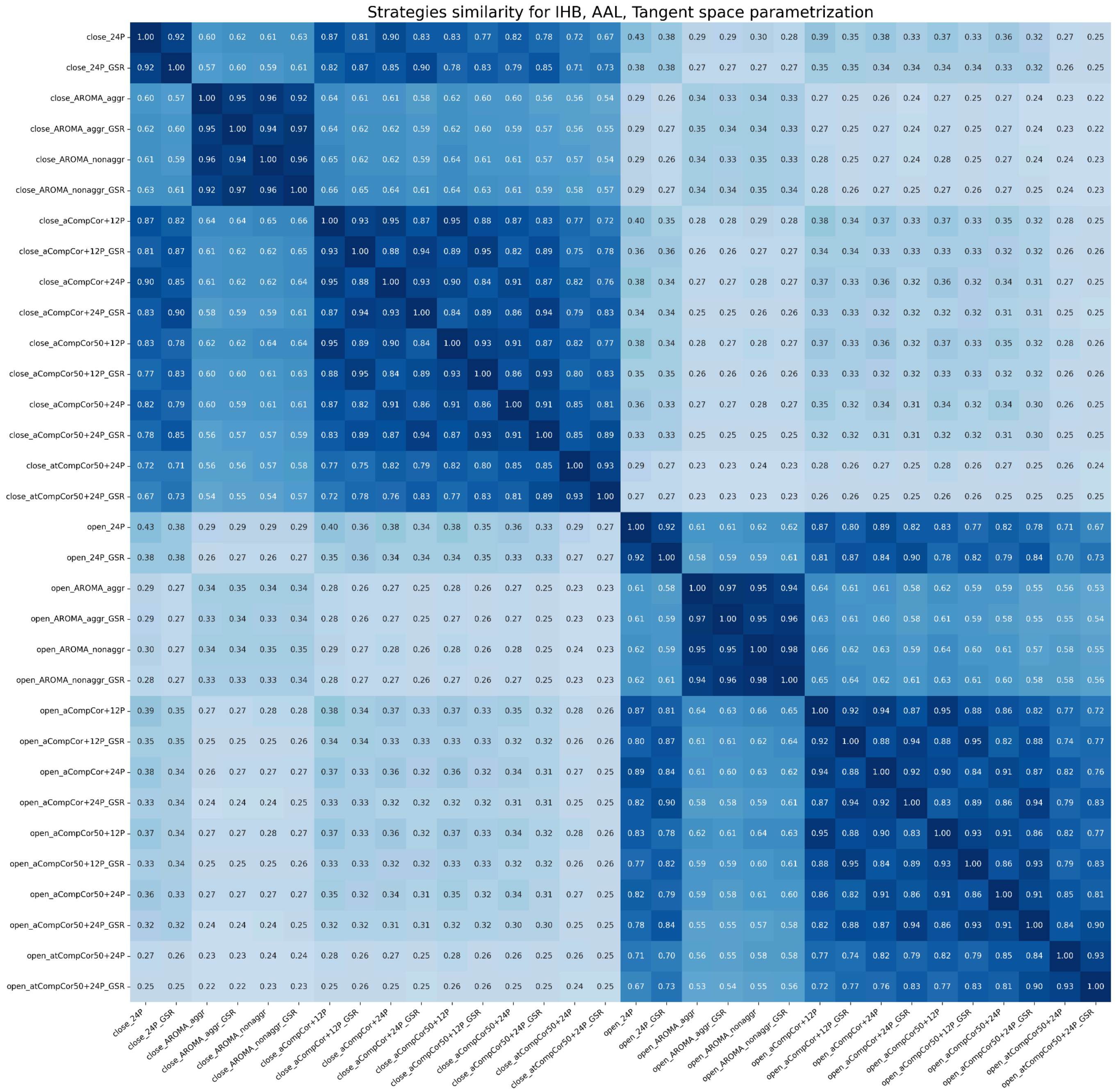

Figure S24.

Similarity between signals of both EO and EC states denoised using different strategies for the IHB dataset, the AAL atlas, tangent space FC. The heatmap shows similarity for one FC measure. The color range is from white (0, low similarity) to dark blue (1, high similarity). Mean values across subjects are presented.

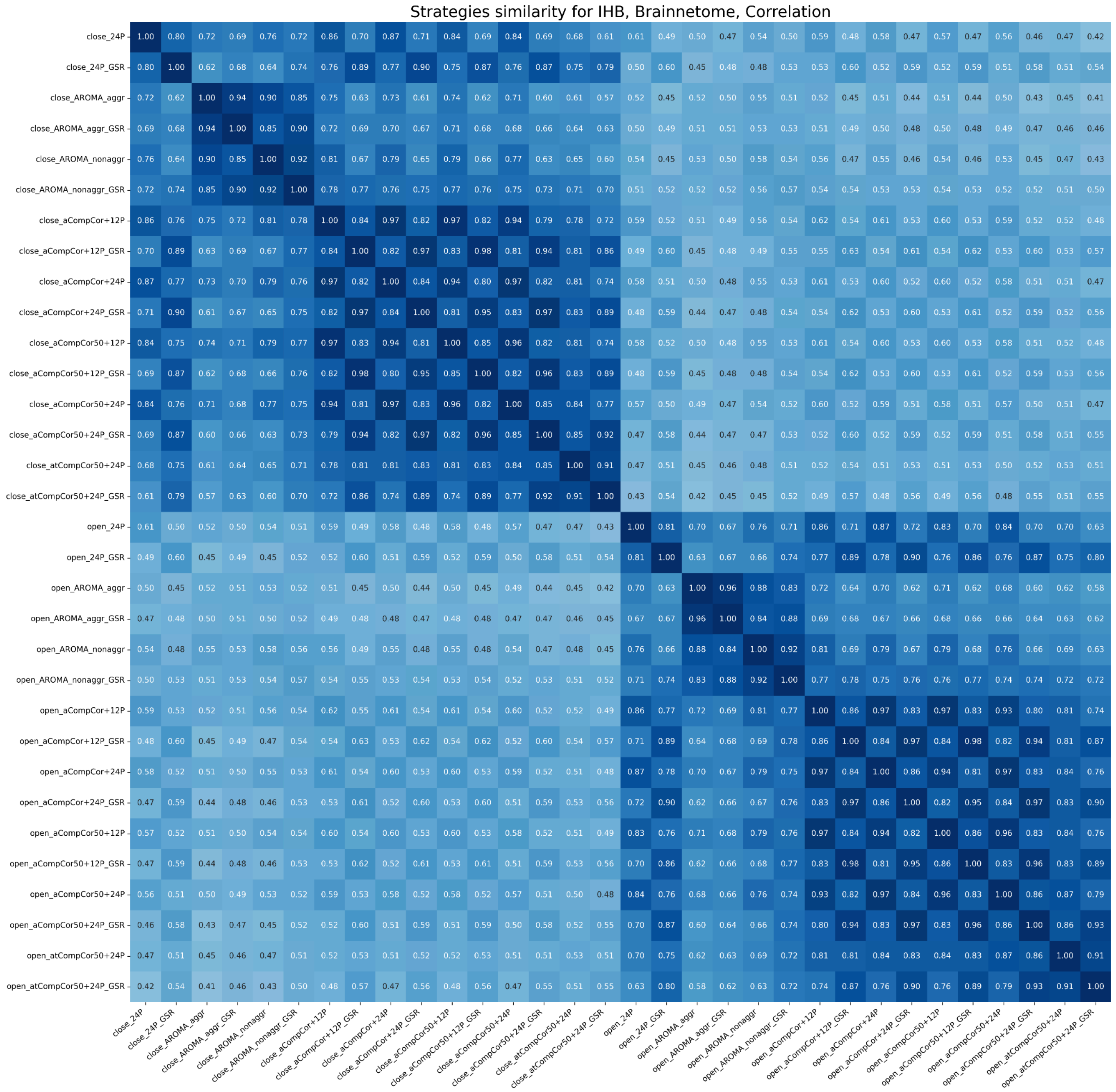

Figure S25.

Similarity between signals of both EO and EC states denoised using different strategies for the IHB dataset, the Brainnetome atlas, correlation FC. The heatmap shows similarity for one FC measure. The color range is from white (0, low similarity) to dark blue (1, high similarity). Mean values across subjects are presented.

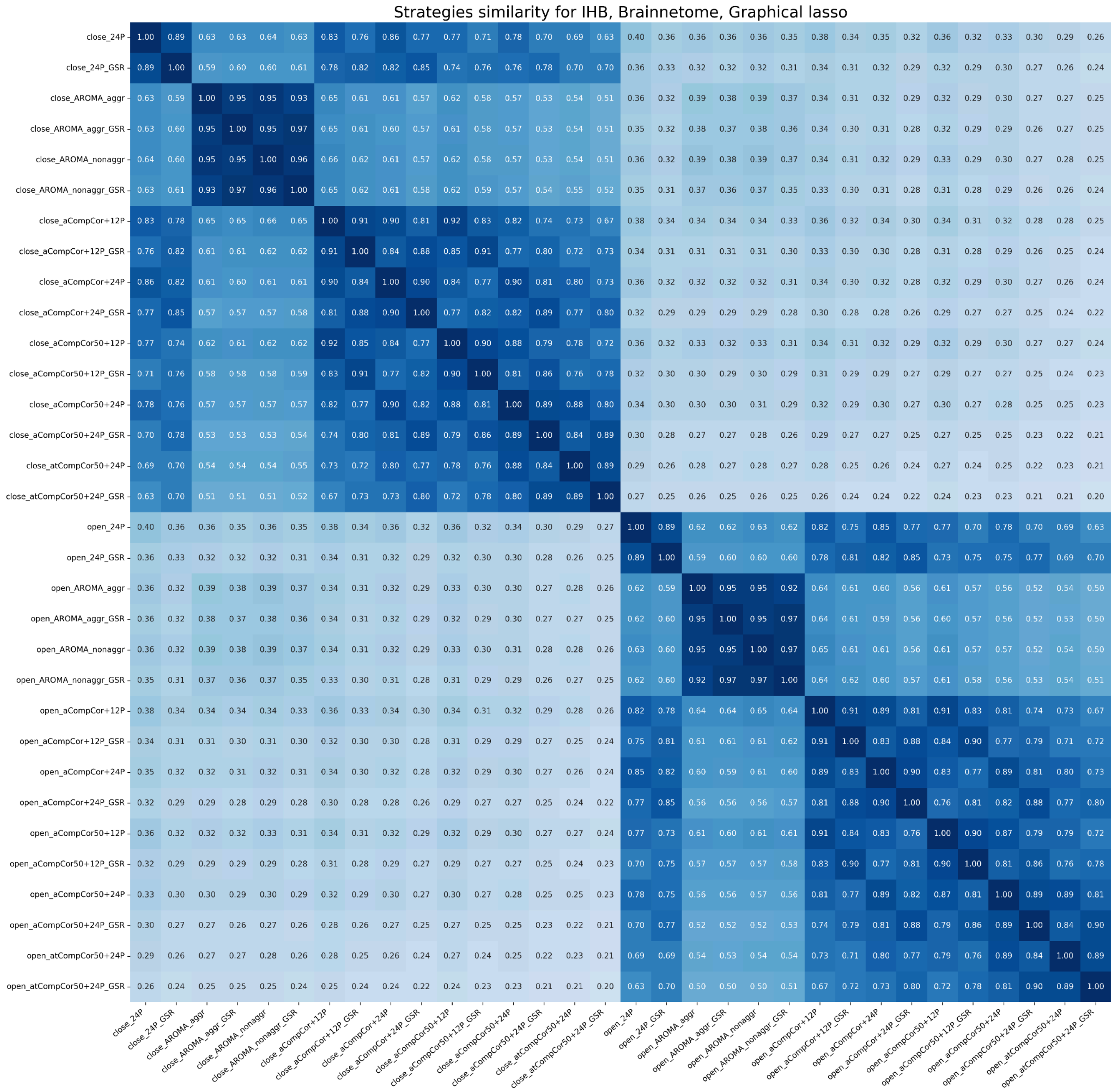

Figure S26.

Similarity between signals of both EO and EC states denoised using different strategies for the IHB dataset, the Brainnetome atlas, glasso FC. The heatmap shows similarity for one FC measure. The color range is from white (0, low similarity) to dark blue (1, high similarity). Mean values across subjects are presented.

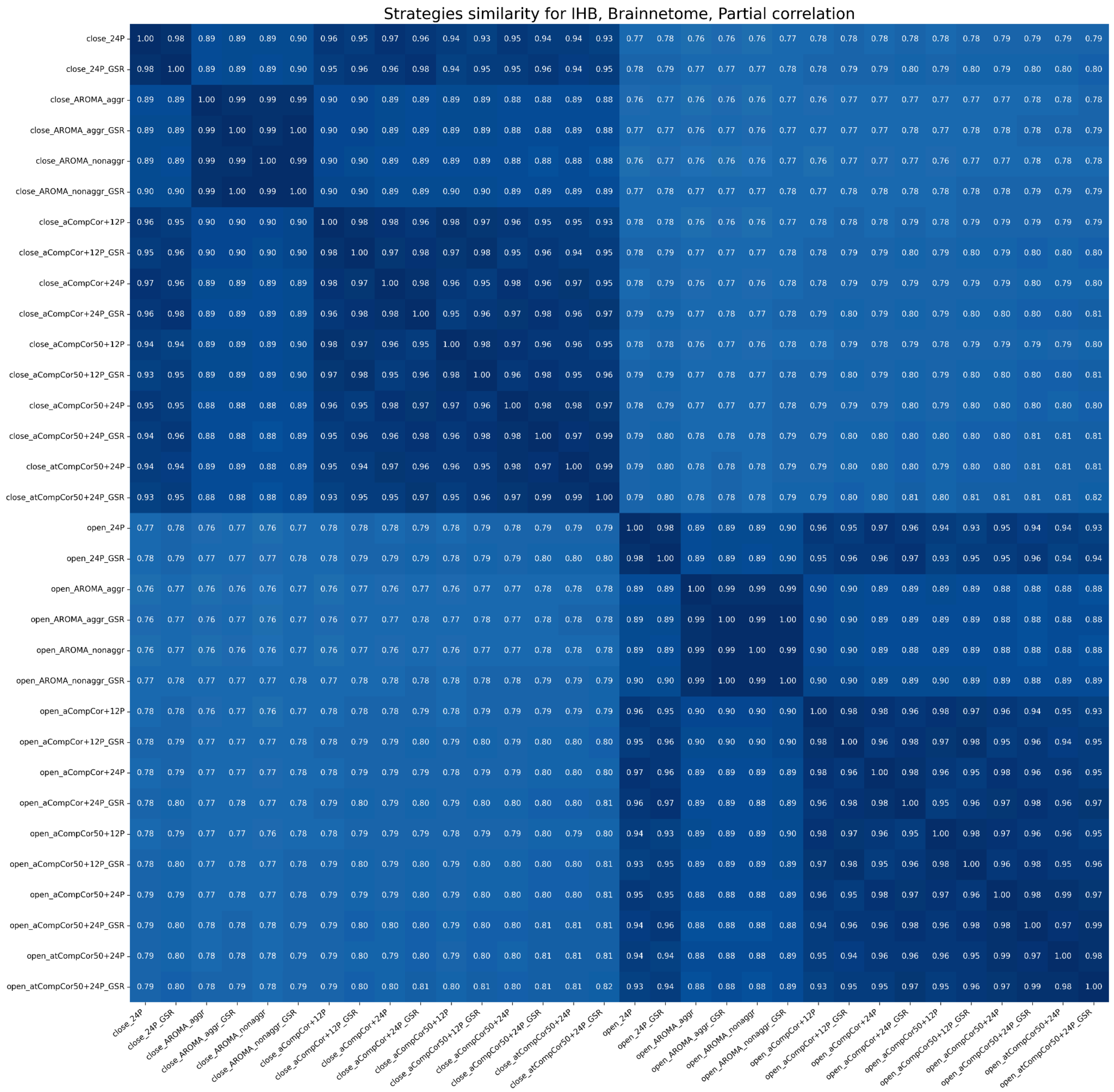

Figure S27.

Similarity between signals of both EO and EC states denoised using different strategies for the IHB dataset, the Brainnetome atlas, partial correlation FC. The heatmap shows similarity for one FC measure. The color range is from white (0, low similarity) to dark blue (1, high similarity). Mean values across subjects are presented.

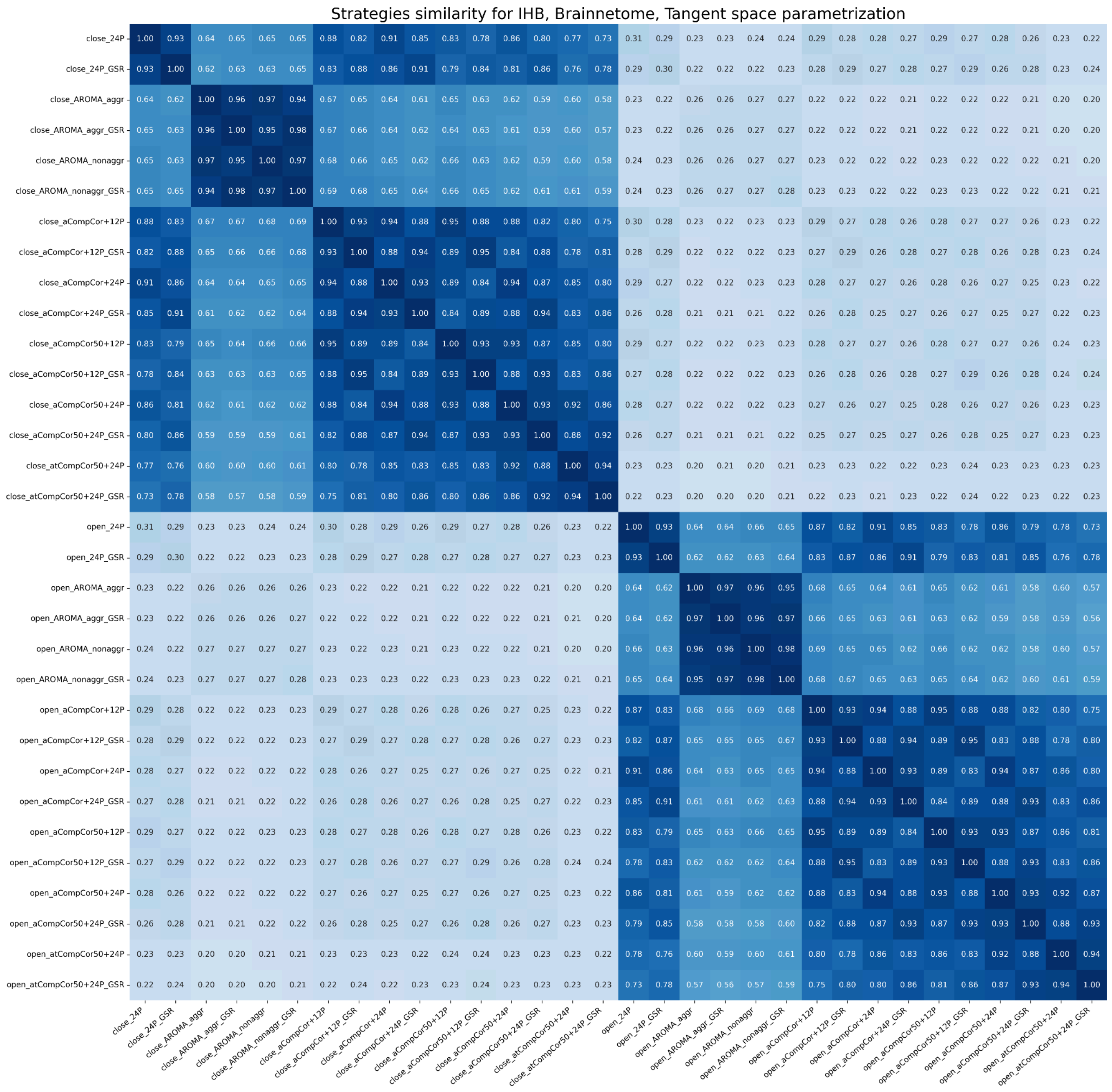

Figure S28.

Similarity between signals of both EO and EC states denoised using different strategies for the IHB dataset, the Brainnetome atlas, tangent space FC. The heatmap shows similarity for one FC measure. The color range is from white (0, low similarity) to dark blue (1, high similarity). Mean values across subjects are presented.

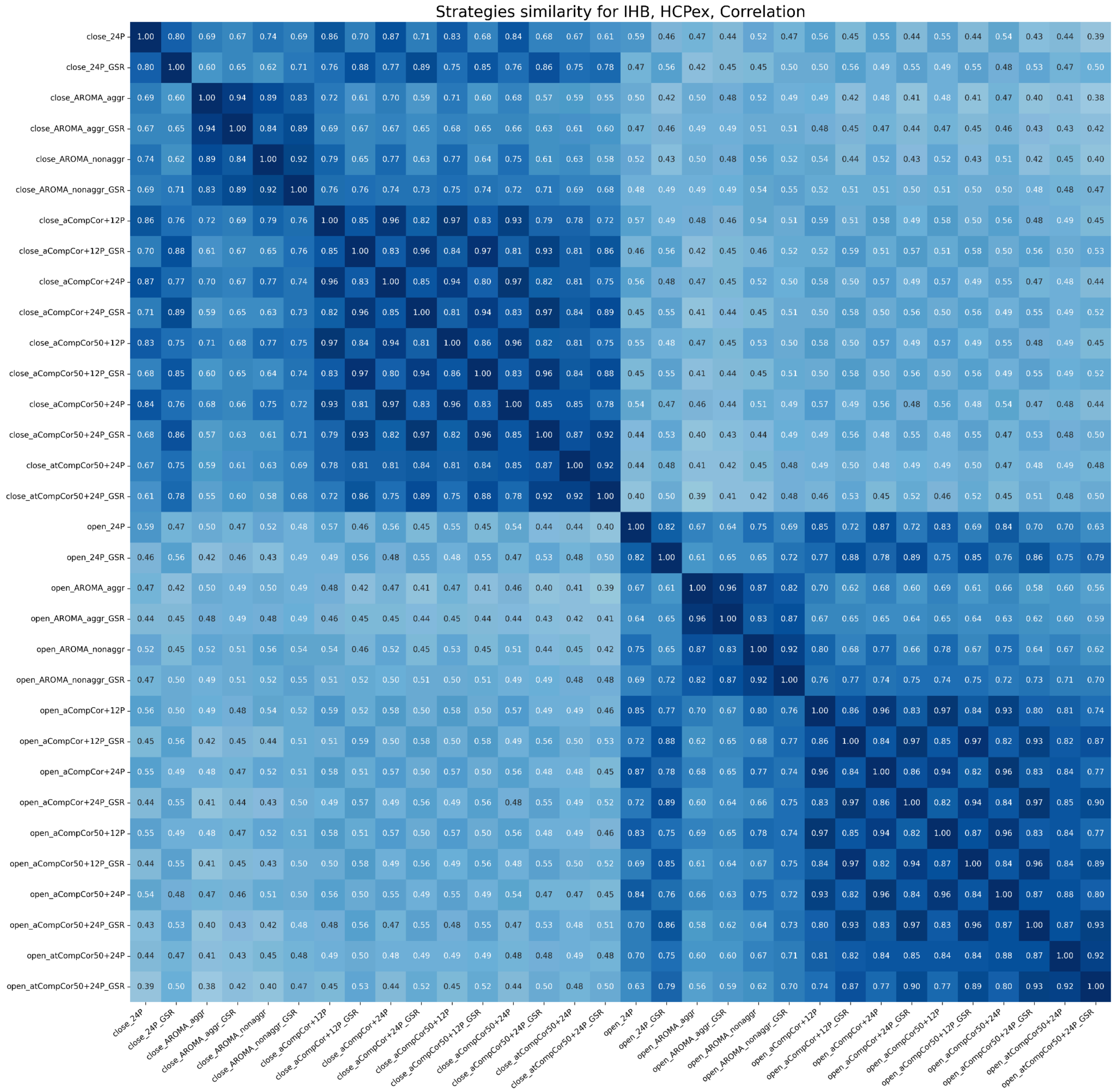

Figure S29.

Similarity between signals of both EO and EC states denoised using different strategies for the IHB dataset, the HCPex atlas, correlation FC. The heatmap shows similarity for one FC measure. The color range is from white (0, low similarity) to dark blue (1, high similarity). Mean values across subjects are presented.

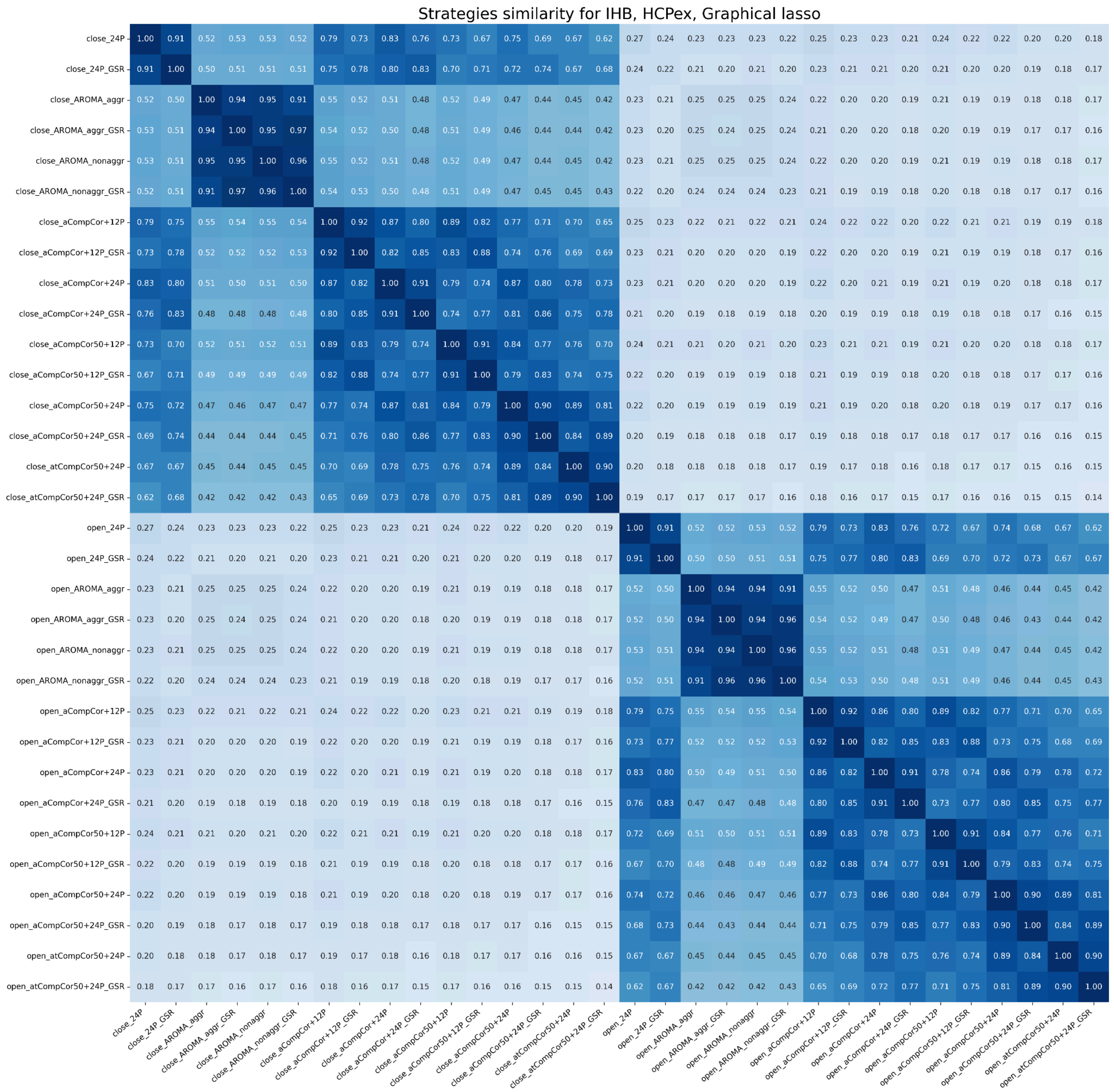

Figure S30.

Similarity between signals of both EO and EC states denoised using different strategies for the IHB dataset, the HCPex atlas, glasso FC. The heatmap shows similarity for one FC measure. The color range is from white (0, low similarity) to dark blue (1, high similarity). Mean values across subjects are presented.

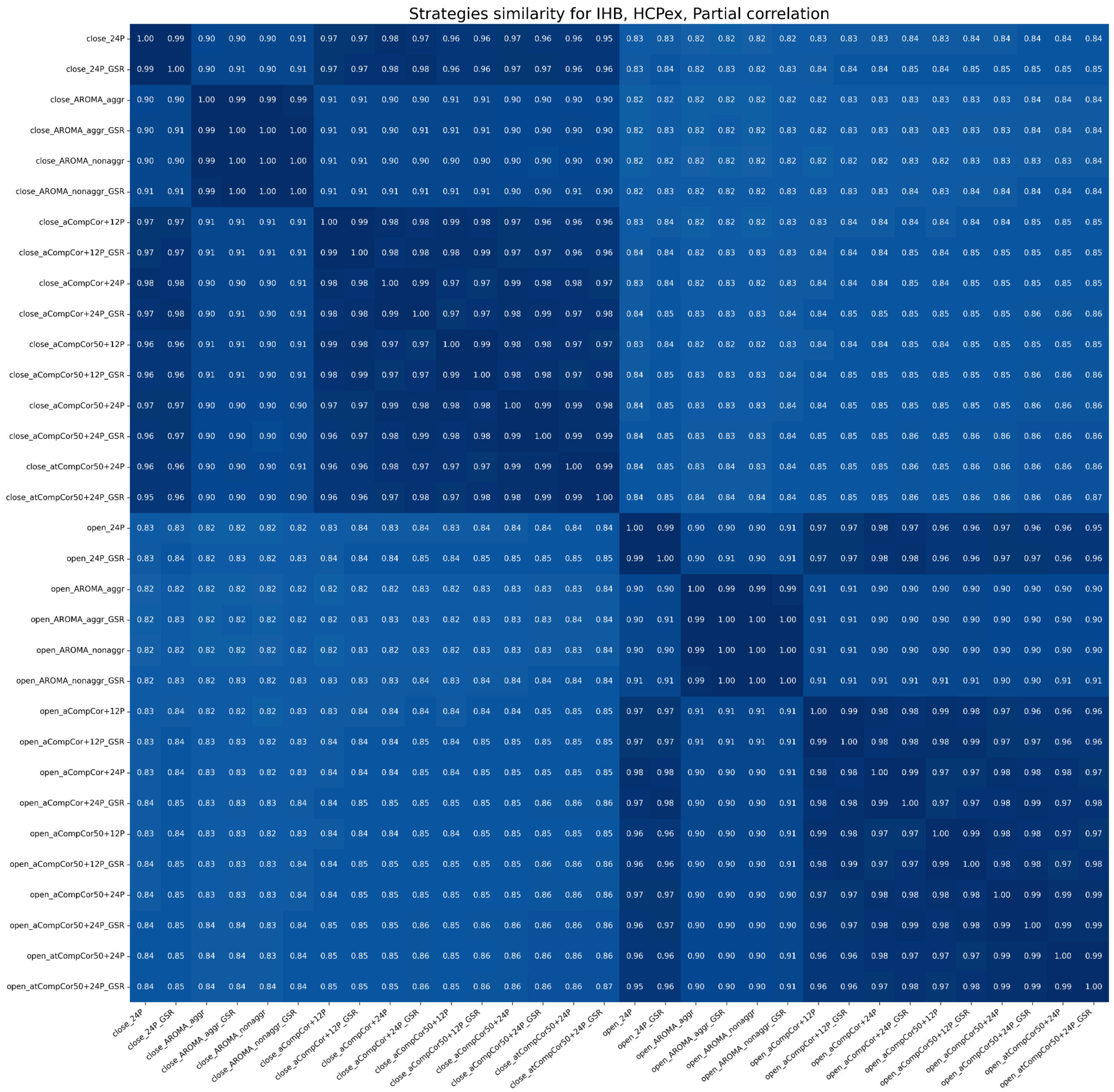

Figure S31.

Similarity between signals of both EO and EC states denoised using different strategies for the IHB dataset, the HCPex atlas, partial correlation FC. The heatmap shows similarity for one FC measure. The color range is from white (0, low similarity) to dark blue (1, high similarity). Mean values across subjects are presented.

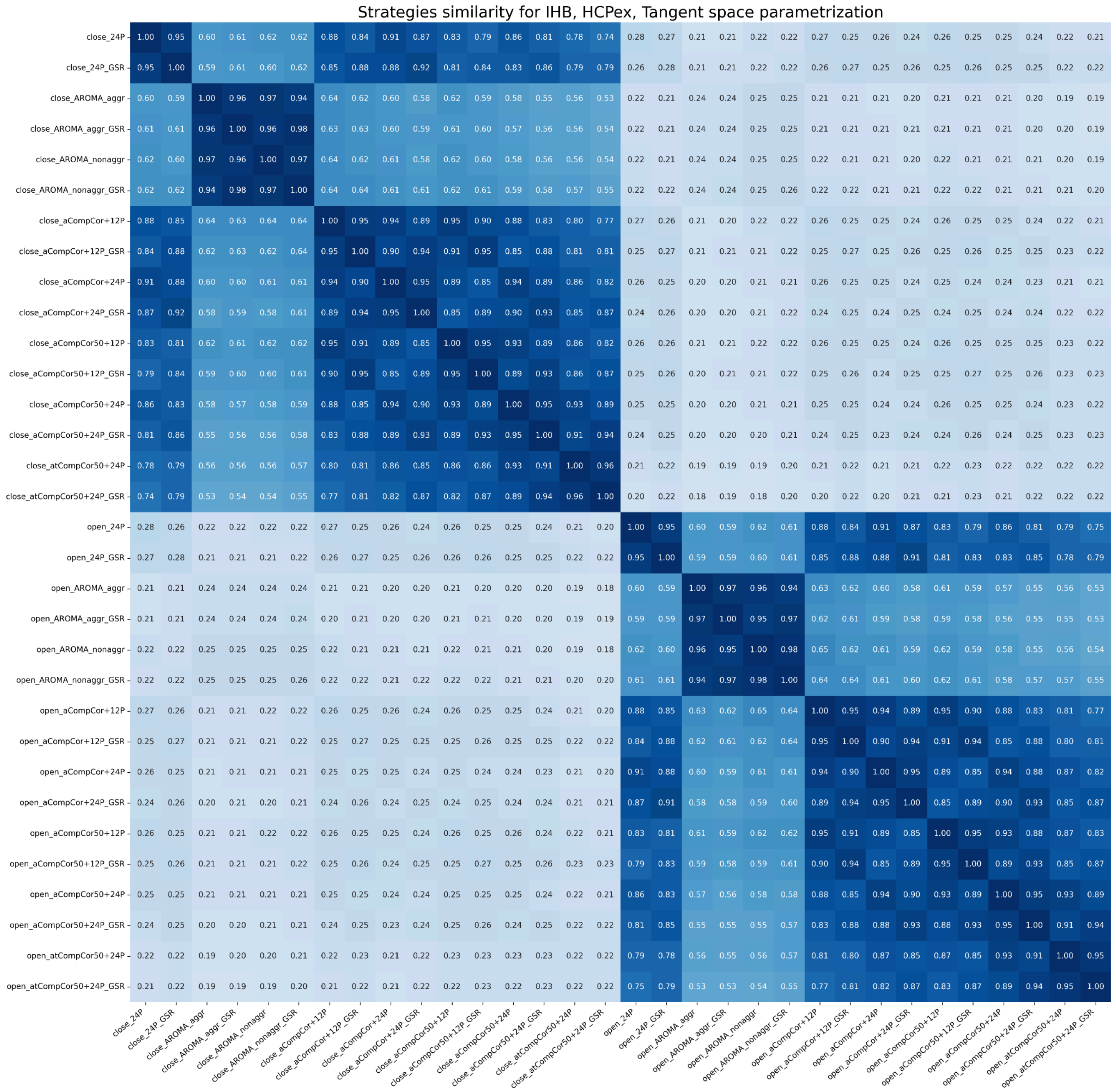

Figure S32.

Similarity between signals of both EO and EC states denoised using different strategies for the IHB dataset, the HCPex atlas, tangent space FC. The heatmap shows similarity for one FC measure. The color range is from white (0, low similarity) to dark blue (1, high similarity). Mean values across subjects are presented.

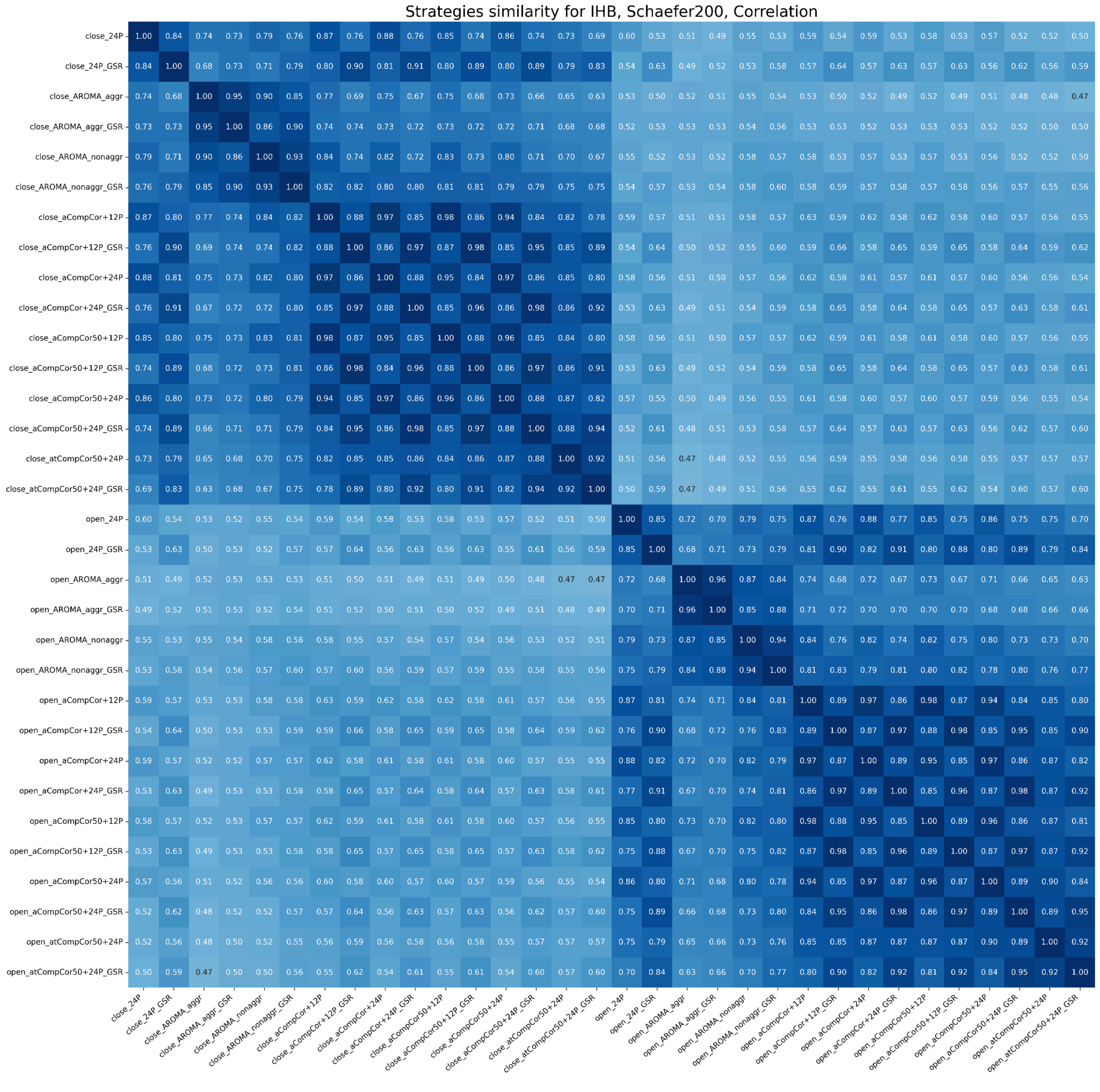

Figure S33.

Similarity between signals of both EO and EC states denoised using different strategies for the IHB dataset, the Schaefer200 atlas, correlation FC. The heatmap shows similarity for one FC measure. The color range is from white (0, low similarity) to dark blue (1, high similarity). Mean values across subjects are presented.

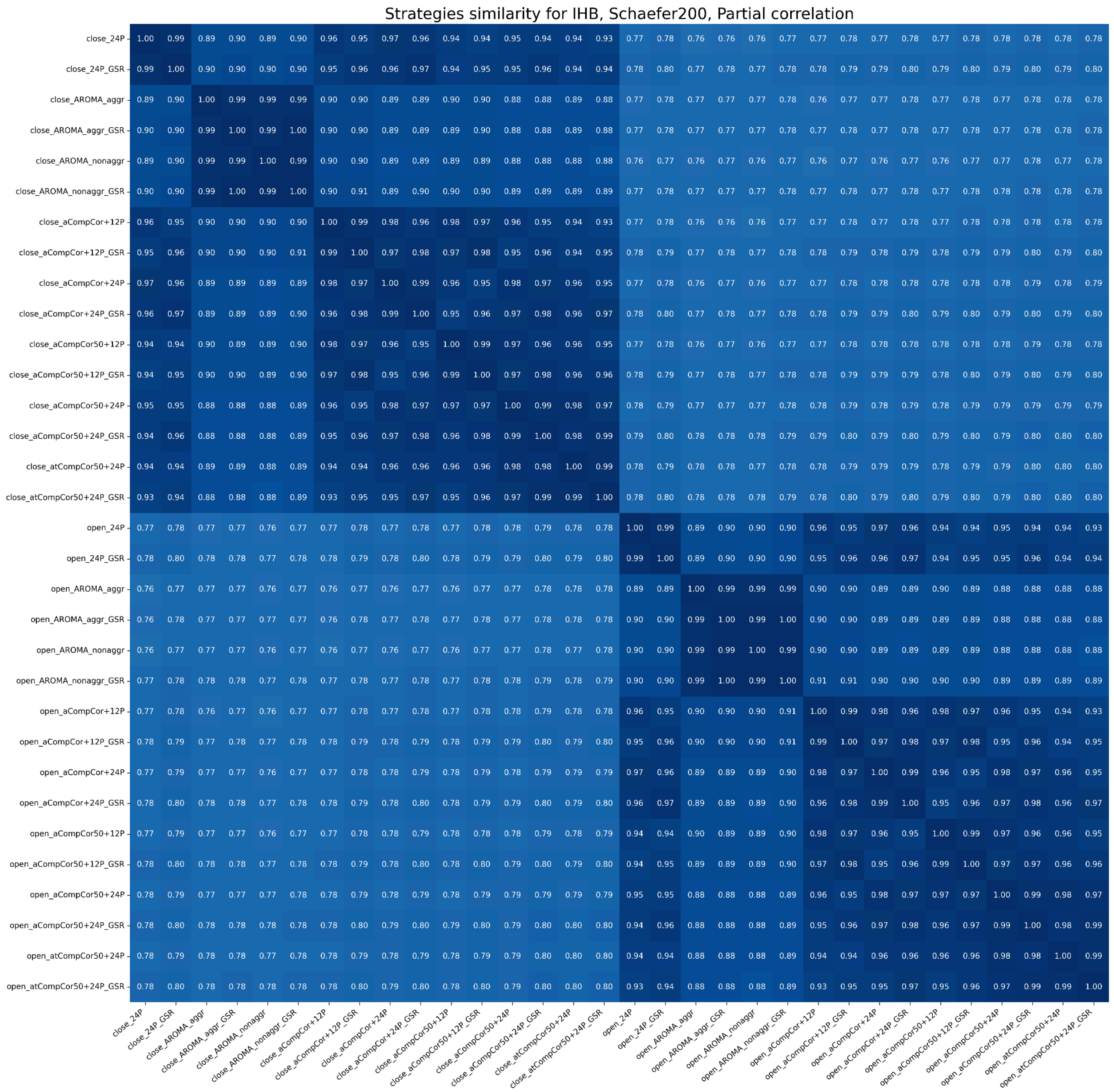

Figure S34.

Similarity between signals of both EO and EC states denoised using different strategies for the IHB dataset, the Schaefer200 atlas, partial correlation FC. The heatmap shows similarity for one FC measure. The color range is from white (0, low similarity) to dark blue (1, high similarity). Mean values across subjects are presented.

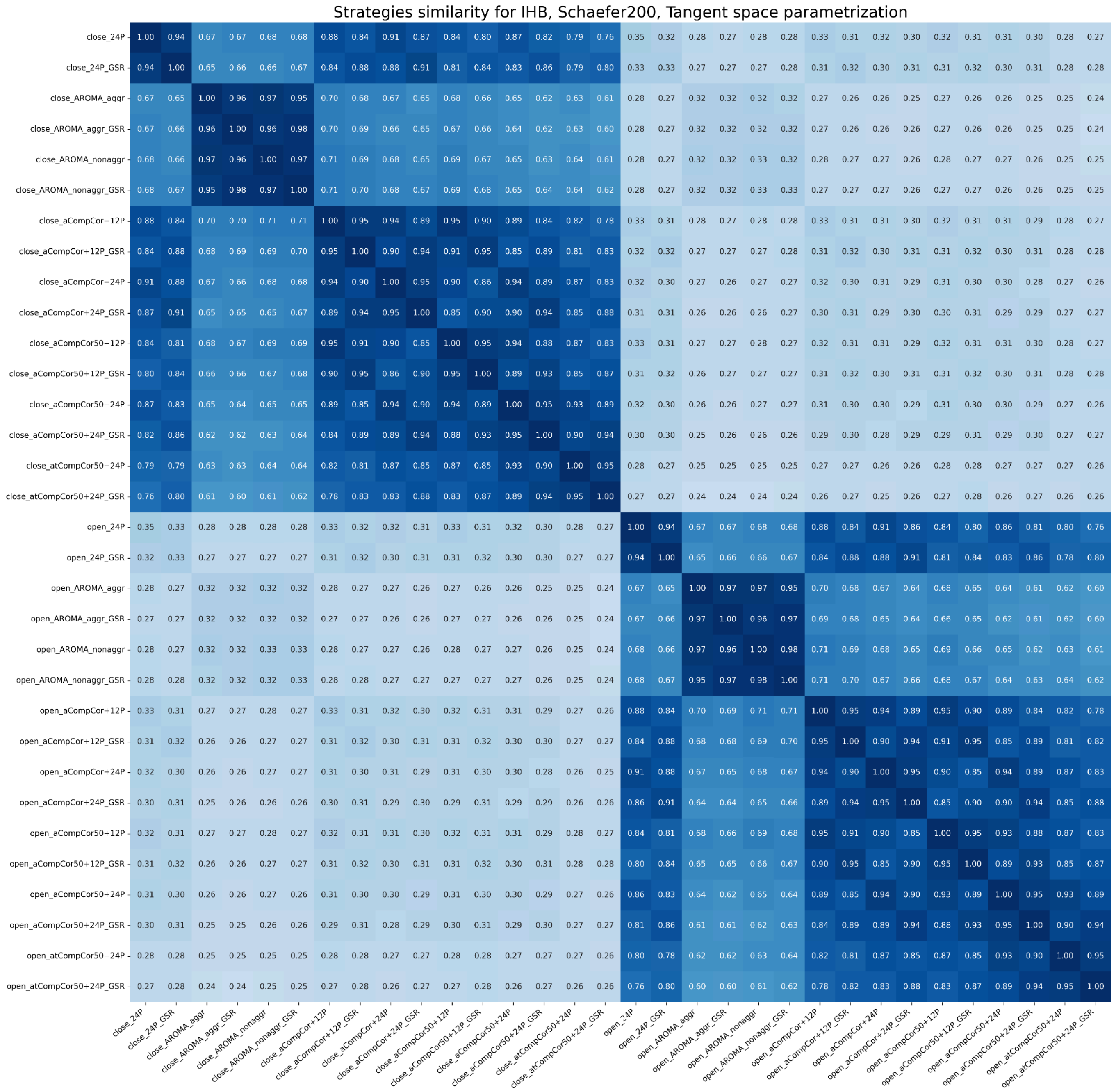

Figure S35.

Similarity between signals of both EO and EC states denoised using different strategies for the IHB dataset, the Schaefer200 atlas, tangent space FC. The heatmap shows similarity for one FC measure. The color range is from white (0, low similarity) to dark blue (1, high similarity). Mean values across subjects are presented.

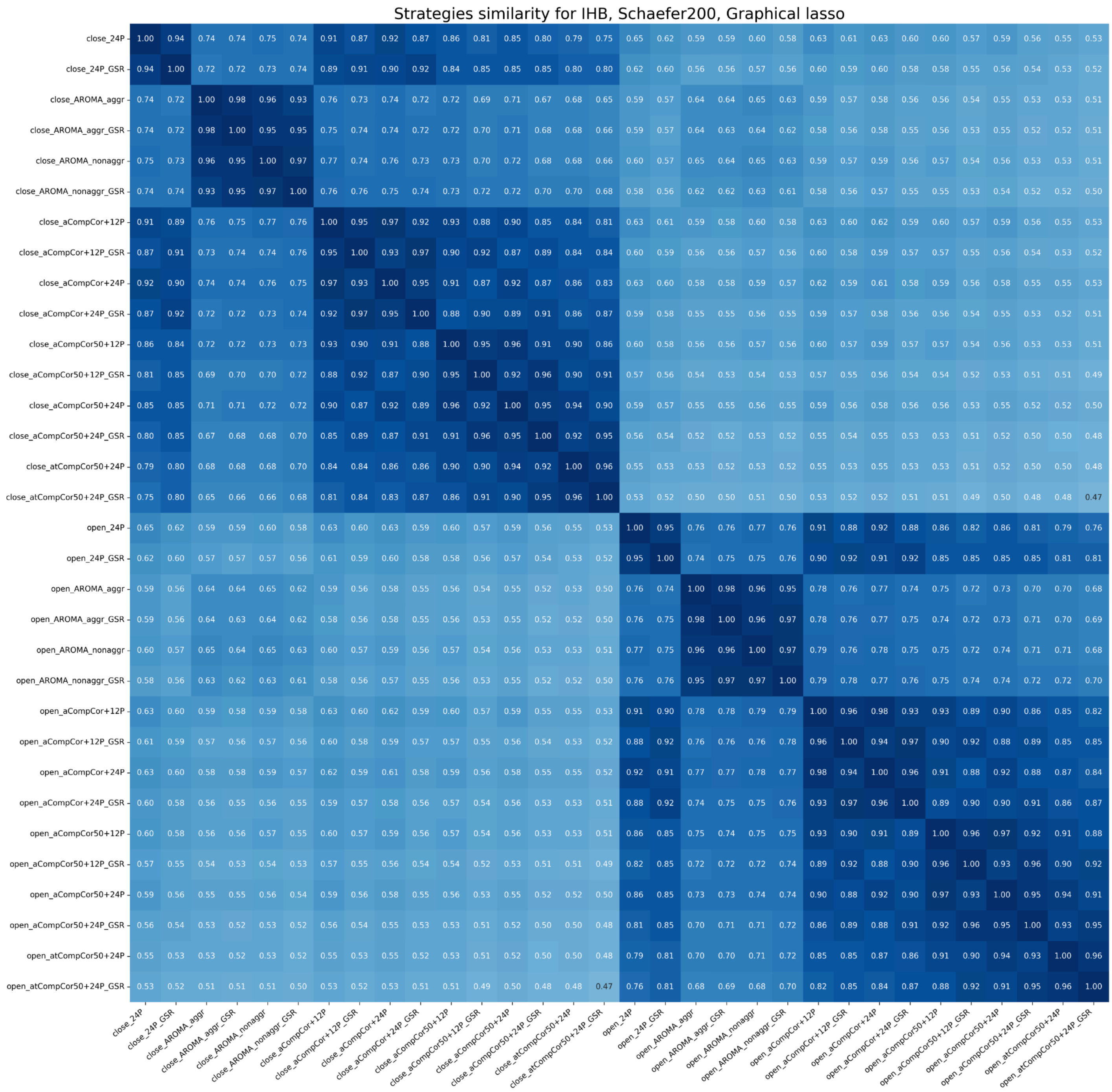

Figure S36.

Similarity between signals of both EO and EC states denoised using different strategies for the IHB dataset, the Schaefer200 atlas, glasso FC. The heatmap shows similarity for one FC measure. The color range is from white (0, low similarity) to dark blue (1, high similarity). Mean values across subjects are presented.
